# Characteristics Associated with Tumor Development in Individuals Diagnosed with Beckwith–Wiedemann Spectrum: Novel Tumor-(epi)Genotype-Phenotype Associations in the BWSp Population

**DOI:** 10.3390/genes12111839

**Published:** 2021-11-21

**Authors:** Kelly A. Duffy, Kelly D. Getz, Evan R. Hathaway, Mallory E. Byrne, Suzanne P. MacFarland, Jennifer M. Kalish

**Affiliations:** 1 Division of Human Genetics, Children’s Hospital of Philadelphia, Philadelphia, PA 19104, USA; duffyka@chop.edu (K.A.D.); hathawaye@chop.edu (E.R.H.); byrneme@chop.edu (M.E.B.); 2 Department of Biostatistics and Epidemiology, Perelman School of Medicine, Philadelphia, PA 19104, USA; getzk@chop.edu; 3 Department of Pediatrics, Perelman School of Medicine, Philadelphia, PA 19104, USA; macfarlands@chop.edu; 4 Division of Oncology, Children’s Hospital of Philadelphia, Philadelphia, PA 19104, USA; 5 Center for Childhood Cancer Research, Children’s Hospital of Philadelphia, Philadelphia, PA 19104, USA; 6 Department of Genetics, Perelman School of Medicine, Philadelphia, PA 19104, USA

**Keywords:** Beckwith–Wiedemann Syndrome, Beckwith–Wiedemann Spectrum, cancer predisposition, tumor screening, hepatoblastoma, genotype-phenotype, multifactorial diseases, environmental stimuli, personalized medicine, phenotypic heterogeneity

## Abstract

Beckwith–Wiedemann Spectrum (BWSp) is the most common epigenetic childhood cancer predisposition disorder. BWSp is caused by (epi)genetic changes affecting the BWS critical region on chromosome 11p15. Clinically, BWSp represents complex molecular and phenotypic heterogeneity resulting in a range of presentations from Classic BWS to milder features. The previously reported tumor risk based on Classic BWS cohorts is 8–10% and routine tumor screening has been recommended. This work investigated the tumor risk and correlation with phenotype within a cohort of patients from Classic BWS to BWSp using a mixed-methods approach to explore phenotype and epigenotype profiles associated with tumor development through statistical analyses with post-hoc retrospective case series review. We demonstrated that tumor risk across BWSp differs from Classic BWS and that certain phenotypic features are associated with specific epigenetic causes; nephromegaly and/or hyperinsulinism appear associated with cancer in some patients. We also demonstrated that prenatal and perinatal factors that are not currently part of the BWSp classification may factor into tumor risk. Additionally, blood testing results are not necessarily synonymous with tissue testing results. Together, it appears that the current understanding from Classic BWS of (epi)genetics and phenotype correlations with tumors is not represented in the BWSp. Further study is needed in this complex population.

## 1. Introduction

Beckwith–Wiedemann syndrome (BWS; OMIM #130650) is the most common epigenetic overgrowth disorder in children, affecting approximately 1 in 10,000 live births and is caused by (epi)genetic mechanisms involving chromosome 11p15.5 [1]. The disorder leads to a variety of clinical manifestations with heterogeneous individual phenotypes, which prompted the reclassification of BWS from a syndrome to a spectrum (BWSp) by a recent international consensus group (ICG-BWSp) [1]. Within BWSp, patients can be affected by isolated lateralized overgrowth (ILO; previously hemihypertrophy/hemihyperplasia) in addition to atypical and classic BWS phenotypes [1]. To aide in clinical and molecular diagnosis, the BWSp-ICG introduced a diagnostic scoring system for patients and stratified common clinical features into cardinal and suggestive features, based on the likelihood of the feature leading to a BWSp diagnosis. A common concern in patients affected by BWSp is predisposition for embryonal tumor development, with approximately 8% of all patients developing a tumor [1]. As a result, identification of patients who may experience a higher risk for tumor development within the BWSp population represents an important goal in guiding clinical care.

A diagnosis of BWSp can be first determined clinically based on features observed and approximately 80–85% of patients have an underlying molecular diagnosis due to an alteration identified in the BWS critical region located on chromosome 11p15 [1]. The three most common molecular subtypes of BWS involve methylation abnormalities leading to a change in expression of the imprinted genes located in one or both of the differentially methylated regions (DMRs) and include loss of methylation at *KCNQ1OT1*:TSS-DMR (IC2 LOM, ~50% of patients); gain of methylation at *H19/IGF2*:IG-DMR (IC1 GOM; 5–10% of patients); and paternal uniparental isodisomy of chromosome 11 (pUPD11; ~20% of patients), which leads to both IC1 GOM and IC2 LOM [1]. Additional rarer molecular causes include chromosome rearrangements of this region, deletions, or duplications of the DMR, or mutations in one of the genes in the region, *CDKN1C* [1].

Consistent evidence has led to (epi)genotype-phenotype correlations, in which clinical features and cancer risk appear to be associated with the DMR(s) altered. Patients affected by pUPD11 and IC1 GOM have a significantly increased cancer risk compared to patients with IC2 LOM [1]. Patients affected by IC1 GOM tend to develop Wilms tumor (WT) while patients affected by IC2 LOM tend to develop hepatoblastoma (HB) and other rarer cancers such as rhabdomyosarcoma [1]. Patients affected by pUPD11 are at risk for development of HB, WT and adrenal tumors [1], likely due to the fact that both IC1 GOM and IC2 LOM occurs within this subtype. In regard to other clinical features, patients with IC2 LOM are more commonly affected by macroglossia, omphalocele, ear creases/pits and nevus simplex [2,3]; and it has been suggested that the BWSp-ICG scoring system is best suited to identify these patients [4]. The phenotype of IC1 GOM and pUPD11 is less specific, with common associations including organomegaly and lateralized overgrowth (LO), which can be isolated (ILO) in some patients [1]. Mussa et al. [3] previously proposed the concept of phenotypic profiles that are characteristic among different BWS molecular subgroups, although noted that there is some degree of clinical overlap between these profiles.

Although consistent (epi)genotype-phenotype correlations have established the molecular subtypes associated with increased cancer risk within BWS, specific clinical features associated with cancer in the BWS population are less understood. Previous investigations have demonstrated that patients affected by ILO and LO have an increased risk and some studies have established an association with organomegaly, mainly nephromegaly and WT risk [3,5]. The importance of (epi)genotyping to determine tumor screening protocols in patients with BWS was first suggested by Bliek, Gicquel [6] and was subsequently supported by a number of other groups [2,7,8]. As a result, the BWSp-ICG did not include the IC2 LOM subgroup within their tumor surveillance recommendations, due to the lower tumor risk that patients experience in comparison to the other subgroups [1]. In contrast, the American Association for Cancer Research (AACR) recommended that all patients affected by BWS (including IC2 LOM) should receive tumor surveillance, due to the benefits associated with earlier cancer diagnosis and a different threshold for acceptable risk [9]. Substantial evidence exists to support the role of HB and WT screening programs within BWSp and other cancer predisposition disorders [10,11,12,13], with consideration for a similar AFP screening for populations at risk for HB [14]. Literature reported after the spectrum of BWSp was established has included a number of case series and case reports of patients affected by IC2 LOM and tumor development [15,16], with reconsideration of the tumor risk in patients with IC2 LOM [12,17]. However, other groups have maintained that the IC2 LOM epigenotype profile does not create a significant increased risk to warrant tumor screening [18].

In the present study, we performed an epidemiological descriptive population study to explore the (epi)genotypes, phenotypes and other population characteristics associated with history of tumor development in a large cohort of patients representing the full Beckwith-Wiedemann Spectrum population. Primary objectives included establishing tumor development rates and statistical exploratory analyses to establish phenotype and/or epigenotype-phenotype profiles associated with tumor history. Secondary objectives included a selected case series for retrospective record review to clarify findings and observations derived from the exploratory descriptive population analyses. We additionally sought to characterize the 11p15 epigenotype profiles detected through clinical laboratory methods in blood, tissue and tumor samples collected from patients within the BWSp population. Our results suggest that 11p15 mosaicism in blood is common, as is tissue mosaicism occurring within internal organ populations of patients. While certain BWSp phenotypes may be associated with tumor development, the complex clinical and molecular heterogeneity characterized in this population suggests that blood testing and external BWS features cannot provide sufficient evidence to support epigenotype stratification to the tumor screening protocol.

## 2. Materials and Methods

We utilized the dataset created for our 2019 BWS Registry cohort [4] and selected patients with a confirmed molecular diagnosis of BWSp due to IC1 GOM, IC2 LOM, or pUPD11 (positive in blood and/or tissue sample). Patients lacking tumor development outcome data and phenotype information relevant to the variables defined for the present study objectives were excluded. This dataset was utilized for the statistical analyses portion of the study.

### 2.1. Description of BWS Registry and Data Collection Methods

The methodology for the BWS Registry database has been published previously [4], with replication of methodology applied to other BWSp cohorts [19,20,21]. A detailed description of the methodology applied for this study is available in Supplementary Appendix A; however, in brief, the BWS Registry collects longitudinal information about patients with a clinical or molecular diagnosis of BWSp through medical records and patient/family interviews as available. Patients and/or their guardian provide consent at time of enrollment in the study (IRB 13-010658).

We reviewed all patients identified through the query and, to the best of our knowledge, none of the patients included in the present study have developed tumors since the March 2019 data abstraction was performed (as of March 2021). No additional information was updated or reviewed. For tumor groups, we initially stratified the data into two categories: Development of any tumor (yes versus no). Additional categories were created to evaluate specific tumor types: Development of HB versus No tumor development; and Development of WT versus No tumor development. In the latter two categories, patients with development of other tumor types not specified by category were excluded (i.e., patients with HB were excluded from the WT categories).

### 2.2. Variable Terms and Description

The following variables were abstracted from the 2019 dataset: BWSp molecular diagnosis characteristics BWSp subtype and positive source of 11p15 abnormality (blood or tissue); demographic characteristics (patient sex and race/ethnicity group); prenatal and birth characteristics (conception type, multiple gestation, polyhydramnios, placentomegaly, premature status and LGA at birth status). Common BWSp features (macroglossia, LO, ear creases/pits, facial nevus simplex) were evaluated as present or not present, while the other BWSp features were evaluated as the overall rate for the category and stratified by specific type of feature: abdominal wall defect (AWD): (omphalocele or minor AWD) and hypoglycemia (transient or hyperinsulinism (HI)). Organomegaly was evaluated overall and by specific enlarged organ type (kidneys, liver and/or spleen). Patients for whom omphalocele or severe hypoglycemia/HI were not documented or reported were considered not to have the feature; other features were considered as missing variables if not explicitly documented. The BWS spectrum groups classified in the 2019 analysis were grouped as the following within the present study: Classic BWS, or Atypical BWSp/ILO. Information regarding tumor development included the type(s) of tumors that developed in the patient at time of last follow-up; these were grouped in the present study as WT, HB, or other embryonal or BWSp-associated tumor categorized through the BWSp scoring system [1]. A list of common abbreviations is available in Supplementary Table A1 and a data dictionary for the variable terms and classifications has been included in Supplementary Table A2.

### 2.3. Population Group Selection and Subgroups Defined

A case-control approach was utilized to assess comparisons within the study population and within subgroups of the study cohort. For all analyses, ‘case’ was defined as history of tumor development and ‘control’ was defined as no history of tumor development at last follow-up. Specific subgroups of patients within the cohort were evaluated separately for the case-control, with the specific groups designated for the tumor-phenotype comparisons summarized in Supplementary Appendix A Section: ‘Exploratory Analyses Methodology’.

We evaluated the cross-sectional distribution of clinical characteristics and features in order to identify potential differences in phenotypic presentation between groups that would be diagnosed prior to tumor development. Given the constellation of clinical features that can present in combination, we did not aim to identify independent risk factors for this study.

### 2.4. Statistical Analysis

Descriptive statistics were performed on all variables to summarize the frequency of clinical characteristics, BWSp features and tumor rate within the study population. Frequencies were evaluated as the number of patients with the characteristic present (or affected by feature) compared to the total number of patients with evaluable data for each variable; patients with missing data were not included within each comparison. Univariate comparisons were performed to evaluate the frequency of characteristics/features between the case-control groups to evaluate whether significant associations or trends were present between groups that could inform tumor-phenotype profiles. Categorical variables were compared with Pearson chi-square and nominal variables were compared using Fisher’s Exact; column proportion (z-testing) with Bonferroni correction methods was performed for all comparisons to identify differences at the *p* < 0.05 level that would suggest a potential trend in the data and/or clarify differences within groups. Statistical significance was set at *p* < 0.05 for all comparisons. All statistical analyses were performed using IBM SPSS Statistics Version 26.

### 2.5. Selected Case Series with Retrospective Record Review

A post-hoc retrospective record review was planned to evaluate a selected case series of patients representing tumor development within the population. Additional patients included in the selected case series were identified from the BWSp Research Network, which includes patients recruited through the BWS Registry, patients identified through investigator lists with data collected through exempt medical record review, as well as collaboration with investigators at other institutions. Appropriate data sharing, material transfer agreement and IRB approvals were obtained.

Before the study, we had established inclusion criteria for this case series as patients with (A) IC2 LOM with tumor development and/or (B) HB development. Additional criteria for patient selection and specific variables of interest during the review were determined after the exploratory analyses were performed, with the main objective being to further characterize and describe the relationships established through the exploratory analysis to clarify results. We reviewed the medical records at our institution, those available from outside institutions and information collected from patient/family interviews to extract information variables and categories of interest determined post-hoc exploratory analyses. No additional post-hoc statistical analyses were performed, as these additional data variables were not available for the full study population. Plans for the case series included characterization of patients by tumor type, epigenotype profile, population characteristics and/or BWSp phenotypic features to identify whether any common variables or characteristics were present that could serve as potential variables to explore in association with tumor phenotype profiles in the future. Additional methodology for variable categories designated for this portion of the study is available in Supplementary Appendix A Section: ‘Case Series Review Methodology’.

### 2.6. Methodology for 11.p15 Epigenotype Profiling

We sought to explore 11p15 methylation levels in blood and tissue samples from patients with history of tumor development to establish ‘epigenotype profiles’ associated with cancer in the BWSp population. Clinical molecular laboratories were utilized and specific testing methodology for the epigenotyping included a combination of SNP array at our institution [22,23] and/or 11p15 methylation analysis as previously described [24,25]. In some patients, high-density microarray-based comparative genomic hybridization (aCGH) was performed as previously described [24] to evaluate for small copy number alterations within the 11p15.5 region. The ‘11p15 mosaic burden’ was determined by the calculated fraction of cells with altered level of methylation at IC1 and/or IC2 (methodology described in [24]). A subset of patients had blood testing performed at other laboratories before care at our institution; these results were reviewed and recorded as positive or negative.

To perform the epigenotype profiling, blood and additional tissue samples were collected from these patients during the course of initial BWSp work-up, routine clinical care and/or surgery treatment with appropriate clinical and/or research consent obtained for the collection. The samples collected were submitted for epigenotype profiling in the clinical laboratories (specific testing determined by ICG-BWSp algorithm, clinical suspicion and limitations of sample availability/quality). At time of enrollment in the BWS Registry, participants provide consent for sharing of samples to the BWSp and Growth Disorder Biorepository for storage and genomic analyses. Collaborators in the BWSp Research Network also have the opportunity to submit samples for epigenotype profiling. Additional samples collected from patients recruited through these methods were submitted for epigenotype profiling in the clinical laboratories.

## 3. Results

There were originally 344 patients (87.8% from United States) included in the 2019 BWSp cohort population dataset [4] and 228 were characterized with a diagnosis of IC2 LOM (*n* = 118), IC1 GOM (*n* = 32), or pUPD11 (*n* = 78) and eligible for the study. From these 228 patients, there were 10 excluded for lack of data available for phenotype profiling and/or tumor development history required for the objectives of the current study and three patients from two unrelated families with IC2 LOM were found to have maternal microdeletion leading to the methylation abnormality.

The BWSp Study Population included for analysis consisted of a total of 215 patients with a molecular diagnosis of BWSp due to a methylation abnormality detected in the IC1 and/or IC2 regions (in blood or tissue sample). Approximately 86% of patients were from North America, with 12 distinct countries or regions besides the United States or Canada represented. The original dataset included information collected through March 2019 and confirmation of tumor development status through follow-up in December 2020 was performed prior to exploratory analysis. There were 1426 person-years of follow-up data recorded for the 215 patients in the study cohort (Supplementary Appendix B Table B1).

Additional detailed 6-month follow-up information was available for 116 patients cared for at our institution through September 2021 (>2 years follow-up from initial recorded follow-up in March 2019) and we can confirm no additional ‘typical BWSp’ tumor development occurred within this subset through clinical care follow-up at our institution; during review, we did identify three additional patients with growths resected that were not considered as a malignant tumor (Supplementary Table B1).

### 3.1. Cancer History Characteristics within the BWSp Study Population

A total of 46 primary tumors were recorded in 43 patients in the dataset during the study period and more than 60% of these tumors were considered ‘typical BWSp tumors’ rather than ‘cardinal feature tumor’ (Supplementary Table B2). The rate of cancer/tumor history within the BWSp population was much higher than expected (between 2-3-fold increase overall) and the distribution of cancer types that developed were not always consistent with what was expected based on the BWSp-ICG published data (Table 1). Consistent with previous cohorts, WT represented the most common tumor to develop; however, a relatively higher rate of HB development was observed within our study population (Table 1). All three BWSp (epi)Genotype groups were observed to have higher rates of tumor development compared to the expected number of tumor cases from the published BWSp-ICG rates and assigned risk for each group and cancer type (Table 1).

#### 3.1.1. Rates of Tumor Development within the 11p15 Epigenotype Populations

Several observations unique to each BWSp (epi)Genotype group were also observed: For patients with IC1 GOM and IC2 LOM, the rate of tumor development was nearly 2-fold what was expected. For patients with IC1 GOM about 50% of patients developed a WT, while for patients with IC2 LOM, there were equal rates of WT and HB observed (Table 1). For patients with pUPD11, the rate of WT development was near twice the expected, while the rate of HB was close to 3-fold (Table 1); rates of development of other tumor types were consistent with expected rates.

#### 3.1.2. Rates of Tumor Development in Males and Females with BWSp

The population rates of males and females within the cohort and subgroups are shown in Table 2. There was a slight female predominance overall in the cohort population, with consistent sex-ratios observed within the population groups (overall population and stratified by tumor or no-tumor development). Female patients were more common in the IC1 GOM subgroup (these findings were previously reported [4]. Wilms tumor (WT) was more common in females within the BWSp study population and within the IC1 GOM population; however, similar sex rates between WT and no-tumor were observed within the pUPD11 population (Table 2).

The sex ratios for HB and no-tumor were similar between groups, suggesting no overall sex-related differences; however, within the pUPD11 population, a higher rate of females with HB was observed (Table 2). Taken together, these observations suggest that sex-related differences may occur within the BWSp population and likely differ by both the specific 11p15 (epi) genotype and the specific type of tumor that tends to develop. The observation of male-HB and female-WT within the five patients in the IC2 LOM subgroup may represent a reflection of cohort bias; other published cases and cohorts have described both males and females with IC2 LOM and tumor development. Further large-scale studies evaluating tumors and IC2 LOM within the full BWSp population may yield additional data to clarify observations within this study population.

#### 3.1.3. Rates of Tumor Development Associated with Assisted Reproduction and/or Multiple Gestation Pregnancies in BWSp Study Cohort

We sought to explore the tumor rate within the subgroups: (1) ART conception and (2) multiple gestation pregnancy history (Table 3). Higher tumor rates were observed for multiple gestation rather than ART conception within the full BWSp study cohort and within the IC2 LOM and pUPD11 groups (Table 3). More WT developed in patients with ART history than HB; and a variety of tumor types developed among the patients who were part of a multiple gestation pregnancy (Table 3). The multiple gestation group was stratified by conception type, with WT or HB associated with ART-multiple and HB or NBL associated with naturally conceived multiples (Table 3).

The overall rate of ART conception and/or multiple gestation was infrequent within the IC1 GOM population; however, it appears that these characteristics may provide a larger influence on tumor rate than the other BWSp populations (Table 3). The tumor rate observed for the ART conception group was half the rate observed for the full BWSp study population (Table 3), while a similar rate to the population was observed within the multiple gestation group, suggesting multiple gestation may provide an influence on potential ART-tumor associations.

### 3.2. Epigenotypes, Phenotypes and Characteristics Associated with Tumor Development in BWSp Study Population

We evaluated the rate of clinical features and characteristics between selected groups of patients without tumor development (no-tumor) and patients with history of tumor development (BWSp-tumor population) to explore potential phenotypes associated with cancer within the BWSp Study Cohort populations. We utilize the term ‘tumor phenotype associations’ to describe significant relationships and statistical trends identified in these analyses. The case-control criteria for each analyses set are described in Supplementary Appendix A. The significance of the associations identified for each variable are differentiated by asterisks for the strength of *p*-value demonstrated: * = *p* < 0.05; ** *p* < 0.01; *** *p* < 0.001 (as appropriate). Significant differences identified through column proportion testing are denoted with footnotes in each of the tables.

#### 3.2.1. Tumor Phenotype Associations in Full BWS Spectrum Population

Although not significant, higher rates of LO and organomegaly were observed within the BWSp-tumor population; with nephromegaly demonstrating a potentially stronger association compared to hepatomegaly or splenomegaly (Table 4). Other common BWS features (macroglossia, FNS, ear creases/pits, AWDs and hypoglycemia) were less frequent within the BWSp-tumor population (Table 4), indicating that patients with tumor may present with milder BWS phenotypes.

A lower frequency of IVF/ICSI was associated with BWSp-tumor demonstrated through column proportion testing, with a difference close to statistical significance through chi-square analysis (Table 4). Additional prenatal and birth characteristics did not differ between groups, nor did other phenotype features evaluated (Supplementary Table B3). Blood positive molecular diagnosis was significantly less frequent in the BWSp-tumor group (Table 4), suggesting tissue mosaicism associated with tumor development. 

#### 3.2.2. Tumor Phenotype Associations in BWSp Clinical Spectrum Groups

Given that previous (epi)genotype-phenotype comparisons were done only within Classic BWS cohorts, we divided our cohort between the Classic BWS and Atypical/ILO phenotype groups to evaluate the (epi)genotype-phenotype correlations with tumors across the complete BWS clinical spectrum.

Nephromegaly and premature birth were significantly associated with tumor development within the Classic BWS phenotype population while individuals without tumors were more likely to have history of FNS (Table 5). No other significant tumor associations were established within the Classic BWS population, including macroglossia, LO, AWDs and hypoglycemia (Supplementary Table B4).

In the Atypical BWSp/ILO subgroup, blood positive diagnosis was less frequent within the tumor group (Table 5), similar to findings observed in the overall BWSp study population. Features such as macroglossia and hyperinsulinism (HI) were significantly associated with lack of tumor development; no significant associations were established for LO or organomegaly (Table 5) or other characteristic features within the Atypical BWSp/ILO population (Supplementary Table B5). 

#### 3.2.3. Phenotypes Associated with Lateralized Overgrowth and Tumor Development

A significance difference in the column proportions for each BWSp subtype was demonstrated by chi-square analysis (Table 6), with LO-tumors associated with pUPD11 and IC1 GOM subtypes. Patients with LO and tumor development were less likely to have BWS testing positive in blood (Table 6), indicating that tissue-mosaic 11p15 changes are associated with tumor development within the BWSp-LO population. No other characteristics or BWSp features were associated with LO-tumor development (Supplementary Table B6). Significant characteristics identified (such as macroglossia, hypoglycemia and AWD) were associated with lack of tumor development within the LO cohort (Table 6). Taken together, these results also suggest that individuals with LO and a tumor may present with milder BWS phenotypes.

#### 3.2.4. Phenotypes Associated with Organomegaly and Tumor Development

The tumor rate in (epi)genotype groups with organomegaly were as follows: 46.2% in IC1 GOM (*n* = 6/13), 40.9% in pUPD11 (*n* = 9/22) and 6.5% IC2 LOM (*n* = 2/31); suggesting that organomegaly in the context of IC1 GOM is associated with tumors to a larger extent than organomegaly-IC2 LOM. Tumors in patients with organomegaly were associated with female sex and individuals identifying as mixed race/ethnicity (Table 7).

No other patient characteristics were significantly associated with organomegaly and tumors within the cohort (Supplementary Table B7), including LO (*p* = 1.000) and positive blood molecular diagnosis (*p* = 1.000). No significant associations with specific organs affected (i.e., kidneys, liver, spleen) and tumors were established; however, tumor patients were observed to have higher rates of nephromegaly while hepatomegaly occurred more often in patients without tumors (Table 7).

#### 3.2.5. Phenotypes Associated with Wilms Tumor (WT) in BWSp Population

Patients with BWSp-WT were significantly less likely to have positive molecular blood testing **(**Table 8), indicating tissue-mosaic 11p15 changes are common within this population. Lack of WT development was associated with increased rates of common BWSp features such as macroglossia, AWD, ear creases/pits, FNS and hypoglycemia (Table 8).

The rate of organomegaly did not differ overall between groups, nor did specific organ types; nephromegaly was more common within the BWSp-WT population while both groups had similar rates of hepatomegaly (Table 8). Differences between conception groups was close to significance, with higher rates of natural conception in the BWSp-WT group and higher rate of IVF and/or ICSI within the group without tumor development (Table 8). Other prenatal and demographic characteristics did not differ between groups (Supplementary Table B8).

#### 3.2.6. Phenotypes Associated with Hepatoblastoma (HB) in BWSp Population

Characteristics were compared between patients without a tumor history and those with history of hepatoblastoma (HB) development within the full BWS Spectrum population. Premature birth was significantly associated with BWSp-HB and column proportion testing demonstrated increased rates of LO and ear creases/pits within the HB group. The overall rate of hypoglycemia was similar between groups; however, HB was significantly associated the severe HI phenotype, while patients without tumor history were more likely to have transient hypoglycemia forms (Table 9).

Although not significant, the BWSp-HB group was affected by LGA at birth, omphalocele and/or nephromegaly more often than patients without tumor development; similar rates of hepatomegaly were observed (Table 9). No significant associations were established related to differences in demographic characteristics, conception type, or pregnancy features including placentomegaly and/or polyhydramnios (Supplementary Table B9). All patients with BWSp-HB had the positive 11p15 finding in a blood sample.

### 3.3. Tumor Phenotype Associations within BWSp (11p15) Epigenotype Population Groups

The three (epi)genotype groups within the BWSp population were evaluated as individual populations to explore whether any cancer phenotype profiles could be established within the unique (epi)genotype BWSp subgroups. For each analysis, the BWSp subtype was selected to control for the (epi)genotype group (other two subtypes excluded) and patients were divided into case–control for cancer development history.

#### 3.3.1. Tumor Phenotype Characteristics Associated with IC1 GOM (BWSp)

Most characteristics and phenotypes were comparable between patients with and without cancer within the IC1 GOM subpopulation (Supplementary Table B10); as all cancers that developed within this population were WT or nephroblastomatosis, we considered these results the ‘IC1 GOM-WT phenotype’. Individuals with IC1 GOM-WT were less likely to present with AWD and/or history of polyhydramnios during pregnancy compared to individuals with IC1 GOM without WT development (Supplementary Table B10). Tissue-mosaic *(rather than positive blood)* genotype confirmation was more frequent within the IC1 GOM-WT population (*n* = 6/15, 40.0%) compared to the IC1 GOM population without tumor development (*n* = 1/14, 7.1%), with significant difference between groups demonstrated by column proportion testing (Supplementary Table B10).

#### 3.3.2. Tumor Phenotype Characteristics Associated with IC2 LOM (BWSp)

No significant associations were established within the IC2 LOM subpopulation (Supplementary Table B11). Due to the lower number of individuals with tumors in the IC2 LOM population (*n* = 5), interpretation of these results should be cautioned. Most phenotypic features did not differ between the tumor groups, except for multiple gestation pregnancy. Multiple gestation pregnancy was more frequent in the IC2 LOM tumor group (*n* = 3/5) compared to the no-tumor IC2 LOM group (*n* = 19/103), with a significant difference between groups demonstrated by column proportion testing (Supplementary Table B11) and close to significance by Fisher’s Exact testing (*p* = 0.056).

#### 3.3.3. Tumor Phenotype Characteristics Associated with pUPD11 (BWSp)

Hypoglycemia was the only characteristic with differences demonstrated between patients with and without tumor development within the pUPD11 population (Supplementary Table B12): Significantly higher rates of overall hypoglycemia (*p* = 0.004) and transient hypoglycemia form (*p* = 0.022) were associated with lack of tumor development within the population, while the frequency of HI did not differ between groups (*p* = 0.439). Nephromegaly was observed to be more common within the pUPD11-tumor group (*p* = 0.094) and similar rates were observed for all other characteristics and features between tumor groups (Supplementary Table B12).

#### 3.3.4. Phenotype Characteristics Associated with pUPD11 and WT

No characteristics or features were significantly increased within the pUPD11-WT population to establish WT phenotype associations (Supplementary Table B13). Patients with pUPD11 and WT history were affected by lower rates of common BWS features such as hypoglycemia, facial nevus simplex, AWD and macroglossia (Table 10).

The rate of positive molecular blood testing was lower within the pUPD11-WT population, but not statistically different (Table 10). Similar rates of LO and organomegaly were observed between patients with and without tumor development; however, non-significant differences in the specific organs enlarged within the pUPD11-WT population was observed (Table 10). No other significant differences were demonstrated between groups, including patient sex and diversity groups (Supplementary Table B13).

#### 3.3.5. Phenotype Characteristics Associated with pUPD11 and HB

Within the pUPD11 population, HB development was significantly associated with positive blood testing and premature birth (Table 11); no other demographic or prenatal/birth characteristics differed between groups (Supplementary Table B14). Significant BWSp features associated with HB included macroglossia, omphalocele, ear creases/pits and nephromegaly (Table 11).

All patients with pUPD11-HB and hypoglycemia were affected by the severe form (HI); transient hypoglycemia was significantly more common in the pUPD11 population without tumor history (Table 11). In the context of all patients receiving +blood testing results, the HI trend suggests that this feature is associated with HB in patients with classic BWS phenotypes, rather than tissue-mosaic BWSp-HI. Other phenotypic features did not differ between groups (Supplementary Table B14).

#### 3.3.6. Tumor Phenotype Profiles Established within pUPD11 (BWSp) Population

As similar rates of the tumor types (WT = 11, HB = 9) were present, we evaluated the findings from the pUPD11-tumor phenotype and the findings from the specific cancer types to determine which findings in the general pUPD11 population were likely related to specific cancer type rather than overall tumor development. Other than hypoglycemia phenotype, no tumor-associations were established within the pUPD11 population for the initial analysis. In both the WT and HB analyses, several characteristics and features were found to be associated with either tumor development, or lack of tumor development within this (epi)genotype population, suggesting unique tumor phenotype profiles to WT or HB development within the pUPD11 (BWSp) population.

#### 3.3.7. Summary of Exploratory Analyses Results–Tumor Phenotype Profiles

The association between WT development in patients presenting with LO and milder BWS phenotype characteristics was confirmed in this study; additionally, the association of tumors with LO phenotype and tissue-mosaic 11p15 changes highlights that negative blood testing cannot always provide the ‘positive source’ for a BWSp diagnosis. Several characteristics and phenotypes within the BWSp population appear associated with HB development, which have not been identified previously in cohorts of patients with BWS. Some of these BWSp-HB associated phenotypes differ compared to established HB risk factors in the general population. While an association between prematurity and HB was established within the BWSp population, we expected to find significant associations with other pregnancy characteristics such as polyhydramnios, conception type and/or male sex with the BWSp-HB phenotype. We did not establish an increased rate of IVF and/or ICSI conception with tumor development within our population; however, we observed trends in conception types across the analyses which suggest that ART may provide risk to certain patients within the BWSp population.

### 3.4. Selected Case Series–Emerging Patterns and Epigenotype Profiles Associated with Tumor Development in BWSp Population

The following criteria were set for the selected case series review based on initial planned methods: (A) IC2 LOM with tumor development and/or (B) HB development, with additional criteria determined post-hoc including patients with history of a tumor and one or more of the following characteristics: (C) ART conception, (D) multiple gestation pregnancy and/or (E) HI phenotype.

We identified 18 patients that met criteria from the 2019 BWSp Study Cohort population and additionally identified 8 patients that met criteria from the BWSp Research Network search for a total of 26 patients included in the selected case series (Supplementary Table B15). The full results of this portion of the study are outside the scope; however, we have included the supporting individual patient data in Supplementary Tables B16 and B17. In brief, we collected all available data on fertility, prenatal complications and phenotype, tumor detection history (pre/post BWSp diagnosis) and other characteristics not included within the BWSp scoring criteria. The BWSp clinical score and Spectrum groups were classified for patients not included in the 2019 Study Cohort; we additionally included pathology variables within this scoring system if available or known (excluded in original 2019 analyses). We evaluated the history of each individual to create groups of patients with similar or common characteristics to explore trends in tumor type, BWSp epigenotype profile groups, or additional characteristics within the case series.

#### 3.4.1. Summary of Results from Selected Case Series

The patients identified for the case series were organized into two primary groups: (1) Multiple Gestation and/or ART Conception and History of Cancer Development; (2) Naturally Conceived Singletons with History of Cancer Development. Although we initially set these two groups, we observed overlapping characteristics between some patients that suggested a ‘pattern profile’ may inform of potential increased risk for tumor development within the BWSp population (Supplementary Table B16). These results are preliminary and we could not validate these variables within the full dataset based on the data limitations. We provide observations within text to guide future investigations.

Characteristics identified for variables of interest focused on preconception and prenatal features (Supplementary Table B17). Severe pregnancy complications associated with twinning were observed in four patients with IC2 LOM profiles and history of twin-twin transfusion syndrome (TTTS) or twin reversed arterial perfusion (TRAP) sequence. In non-identical male-male twins, we also observed severe pregnancy issues such as pre-eclampsia toward the end of the pregnancy (Supplementary Table B17). We also evaluated fertility history and ART use along with severe prenatal or birth phenotypes (organs, AWD/GI, GU) and prenatal characteristics such as vanishing twins. After birth we looked at abnormal placental characteristics, classic (milder) BWSp presentation not appreciated until after tumor diagnosis and atypical presentations (difficult to ‘predict’ or diagnose BWSp pre-tumor) with supporting data available in Supplementary Tables B16 and B17.

#### 3.4.2. Epigenotype Profiles Established in Blood Samples from Patients with Tumor Development

Blood samples from 17 individuals with history tumor development had 11p15 epigenotype profiles established through clinical laboratory testing. The methylation percentage detected at the IC2 and IC1 regions and the affected cell fraction for IC2 and/or IC1 burden within the blood sample analyzed are shown in Table 12. We observed a variety of 11p15 cell burden between patients, with some affected by high levels and others affected by more mosaic (or normal) levels within their blood samples (Table 12); patients with higher cell burden tended to receive a diagnosis of BWSp prior to tumor detection through screening. While the BWSp clinical score appears to correlate with the cell burden, it does not appear that the BWSp score or spectrum group classified provide information on potential risk for tumor development within an individual.

Within this cohort, two patients had negative blood profiles established in the same laboratory, in which a positive tissue sample was tested. The burden of 11p15 may inform risk for potential additional tumor development, as we observed some patients within this series with high BWSp cell fractions and severe BWSp clinical scores who have developed multiple primary tumors to date (Table 12); however, we observed other patients with Severe and Classic BWS without additional tumor development. Additional details on the phenotype and epigenotype profiles from individuals with pUPD11 and multiple tumor development are available in Supplementary Table S1 (clinical characteristics) and Supplementary Table S2 (epigenotype and somatic HB tumor testing). In three HB samples collected from two females with pUPD11 detected in blood, there were three unique CTNNB1 variants identified (Supplementary Table S2), representing the only other common genomic abnormality found in the two patients other than pUPD11.

#### 3.4.3. Hepatoblastoma Epigenotype Profiles Associated with Mosaic IC2 LOM

We previously reported mosaic 11p15 associated with WT development within the BWSp population [27] and recently reported a series of patients with tumor presentation and low-level mosaicism detected in blood but in some cases without other clinical features of BWS [15]. Here, we provide additional evidence for mosaicism associated with HB development and mosaic methylation findings between blood and tissue samples collected. The results are summarized in Table 13.

Clinical BWSp testing results and HB tumor samples were available from five patients, with normal liver available from three. In three patients with IC2 LOM in blood, the 11p15 methylation profile in the normal liver and/or HB tumor samples showed discrepant profiles compared to the blood profile established (Table 13). Two with IC2 LOM profiles in blood had additional IC1 GOM occurring in the liver or tumor without involvement of pUPD11 (#04, #06). This suggests that two isolated methylation events occurred within the organ environment.

One patient with normal blood profile was found to have mosaic IC2 LOM in HB sample (#16). The other two patients had consistent IC2 LOM detected in their samples; however, various levels of mosaic IC2 burden were observed (Table 13). We also analyzed a saliva sample from the mother of Patient #01, finding a normal 11p15 methylation profile (Table 13). One of these patients was included in the BWSp Study Cohort (#01) with three additional patients not included within previously published cohorts (personal cases treated at our institution). We additionally performed an analysis of HB sample collected from patient No. 23 previously reported by Fiala et al. (#06 in present study).

Although Patients #01 and #02 presented with similar epigenotype and BWSp phenotypes, the history tumor development history differed with Patient #02 experiencing recurrent HB and Patient #01 without any additional HB or other tumor development through follow-up more than 5.5 years since end of HB treatment (Supplementary Table S3). Both patients had their HB detected through screening with abdominal ultrasound imaging findings correlated with rising α-fetoprotein (AFP) serum levels; Patient #01 was observed to have rapidly increasing AFP values in the weeks between first imaging detection and biopsy/resection (Supplementary Table S3).

#### 3.4.4. Additional Evidence for 11p15 Tissue Mosaicism Associated with Cancer

We identified unpublished epigenotype profiles associated with mosaic IC1 GOM-WT and neuroblastoma (NBL) through the retrospective review. Although these cancer types were outside of the scope of the present study, we are including a summary of findings in text, with supporting evidence available in Supplementary Tables S4 and S5.

Wilms Tumor: We have collected additional evidence for WT development associated with IC1 GOM due to low-level mosaicism in blood and/or tissue samples, with results available in Supplementary Table S4. These patients also represent WT development in the context of ART conception history. Patient #17 was previously reported in (MacFarland, Patient 9). Since this report, we have collected a blood sample, which established a normal 11p15 methylation profile. Additional data collected since initial report are available in Supplementary Table S4. Patient #20 was recently reported with her clinical history [28]; however, her specific epigenotype profile has not been published previously (Supplementary Table S4). Of note, this patient was excluded from the 2019 BWSp Study Cohort for classification of blood negative (no tissue testing) within the dataset.

Neuroblastoma: We identified two patients with neuroblastoma development in the selected case series. Of note, patients with *CDKN1C* mutations (associated with highest risk for NBL development [1]) were not included within the query. Full details about these two individuals are presented in Supplementary Table S5, including epigenotype data demonstrating mosaicism between samples analyzed. Both NBL tumors were diagnosed by incidental CT imaging and showed positive MIBG on additional imaging work-up but only one patient had positive HVA/VMA values, with normal values observed in the other patient (Supplementary Table S5). Patient #21 represents an unreported patient who presented with HI and NBL with concern for BWSp with negative blood and skin testing; however, various levels of 11p15 cell populations were detected in the pancreas and NBL tissues consistent with a BWSp diagnosis (Supplementary Table S5). Both the unaffected and affected pancreas samples were found to have BWSp profiles consistent with pUPD11, with a higher burden within the ‘affected pancreas’ sample compared to the ‘normal pancreas’ (Supplementary Table S5). The NBL sample was found to have nearly all chromosomes affected, while the BWSp methylation profile suggested pUPD11 (Supplementary Table S2). Patient #03 represents a female (MZ triplet) with BWSp due to IC2 LOM who has previously been reported (ID#26 in [26]); however, her cancer history represents a new report. She developed NBL during her NICU treatment course for extreme prematurity (27 3/7 weeks) and severe BWSp phenotypic consequences, including giant omphalocele (entire liver extra-abdominal), macroglossia and nephromegaly; other complications included large cardiac defect (prenatally detected) and a congenital diaphragmatic hernia (CDH) in addition to severe respiratory issues (Supplementary Table S5). The NBL was confirmed through biopsy and spontaneously regressed. Additional evidence for tissue mosaicism was demonstrated by BWS testing of her tongue (collected during TR) and prenatal amniocytes (Supplementary Table S5). Her twin presented with mild BWSp phenotype and positive IC2 LOM detected in blood, representing concordant epigenotypes and discordant phenotypes between the two surviving ‘identical’ twins from original triplet gestion (Supplementary Table S5). The proband’s twin was not included in the cohort study population analyzed due to her discordant less-affected twin status (as previously described [4]); however, this pair was included in a previous twin study [29].

## 4. Discussion

A common concern among both parents and physicians caring for patients with BWS/BWSp is cancer development. To manage personal cancer risk in this population, tumor screening programs have been developed with the goal of detecting tumor growth early to reduce treatment burden and improve patient outcomes [1,9]. Additionally, the BWSp tumor screening program was shown to provide a psychosocial benefit to parents and caregivers [1,9,17,30]. We previously identified that parents were more comfortable with stratified screening programs if they understood their child’s unique risk associated with BWSp subtype [30]; however, many parents, pediatricians or other physicians are not familiar with the complexities of BWSp and differing tumor risk, leading to an uncertain environment within the BWSp community.

Differing medical environments and specific BWSp tumor screening recommendations among groups represent a primary source for confusion within this community. The first international consensus group (ICG) for BWSp was established in March 2017 and provided a standardized clinical scoring criteria and testing algorithm to enhance diagnosis and management [1]. A major limitation of this group was that the recommendations from the ICG-BWSp were derived from historical data, providing a major limitation to apply and generalize the rates to the Beckwith–Wiedemann Spectrum clinical population (as these rates comprised mainly ‘classic BWS’ cohorts). An additional discrepancy in tumor screening guidelines among recommendation groups includes the use of AFP screening [9], with this ‘screening measure/tool’ not included within the ICG-BWSp recommendations, with justification that abdominal U/S would detect a HB [1].

The goal of this study was to explore tumor development rates and characteristics associated with history of tumor development among patients representing the full Beckwith-Wiedemann Spectrum population. Several tumor-phenotype associations were demonstrated within comparisons of the full cohort group (IC1 GOM, IC2 LOM, pUPD11), with trends and associations established related to craniofacial features, AWD and organ abnormalities (enlargement, hypoglycemia, etc.). While we did observe some trends with tumor development history and LO phenotype, it appears that other than isolated LO and WT, this specific phenotypic feature is not as informative as some other patient characteristics. These other characteristics or potential profiles identified were unique to (epi)genotype groups or other phenotype subpopulations, suggesting that a ‘universal risk profile’ cannot be established based on the blood testing results and/or the ICG-designated BWSp features [1]. To further explore potential characteristics that may inform personal tumor risk, we evaluated common clinical characteristics in a selected group of patients with unusual BWSp-tumor associations related to prenatal and diagnosis histories in addition to exploring tissue mosaicism within the BWSp-tumor population. The observations of these patients suggest that characteristics such as history of twinning, severe pregnancy complications and issues involving the GI, GU and kidney systems are common among patients with BWSp and history of tumor development. Furthermore, these characteristics were observed within all three of the 11p15 (epi)genotype groups, which suggests that the 11p15 region (and not a specific epigenotype) and its established cancer predisposition potential is the only characteristic established to date that can inform potential risk.

Weksberg et al. [31] previously suggested that alterations in the telomeric domain (IC1) versus centromeric domain (IC2) may result in distinct tumor predisposition profiles and those imprinting defects can activate distinct genetic pathways for embryonal tumorigenesis. Application of this concept suggests that genes involved in the IC1 region influence risk for WT development [31] and conversely that genes in the IC2 region likely influence risk for HB development [32]. Although the results of our study generally support this from the descriptive rates observed, the unique findings within our selected case series in combination with published evidence of the evolving phenotype of BWSp suggest that HB can occur in patients with isolated IC1 GOM [15,33] and WT can occur in patients with isolated IC2 LOM defects [10,12]. The results from the tissue 11p15 epigenotype profiles collected from liver and kidney samples presented in this study and previously by others suggest that an ‘isolated methylation defect’ detectable in blood (i.e., IC2 LOM or IC1 GOM) may provide the cancer predisposition environment and contribution from a different cell population (i.e., IC1 GOM to blood-IC2 LOM) may provide the influence that ‘tips the scale’ to cancer development versus predisposition.

Patients affected by pUPD11 are at risk for developing both WT and HB [1] and our results provide interesting observations relevant to understanding tumor-phenotypes within BWSp. It appears that HB development may be associated with certain BWSp phenotypic characteristics, at least within the pUPD11 subgroup (as demonstrated through statistical analyses). It has previously been reported that patients affected by pUPD11 and severe phenotypes may have a higher risk for HB development [34,35]. Our exploratory analyses results support this, as increased frequencies of macroglossia, omphalocele and ear creases/pits were found within our analysis of HB phenotype within the pUPD11 subgroup. Interestingly, the majority of these features are commonly associated with the IC2 LOM phenotype (i.e., macroglossia, omphalocele, ear creases/pits) [2,3]. As the centromeric region has been hypothesized to influence HB development [32], these results suggest that pUPD11 patients affected by features associated with the centromeric domain may be more likely to develop HB compared to pUPD11 patients without features commonly associated with this domain. Although craniofacial features were significantly less frequent within certain BWSp-tumor groups in the exploratory analyses, the selected case series demonstrated that at least a subset of patients with WT are affected by mild craniofacial phenotypes, with more subtle features such as ear creases and FNS appreciated more often than overt macroglossia; in addition, ART and twinning may play a role.

The embryogenesis of developmental asymmetries was previously described in the context of epigenetic mosaicism and twinning by Boklage [36] and it was suggested that the body’s directional axes are established within the first few days post-fertilization. During early cell divisions, the cell progeny is determined by a transcriptional factor subsystem and some of these cells later migrate to the gonadal ridges; BWS was included as a potential imprinting syndrome of interest to further explore these theories [36]. Clinical evidence to support these theories in the context of BWSp was provided by Bliek et al. in 2009, suggesting that a shared source of hematopoietic stem cells (HSCs) could serve as an explanation in the context of twins with BWSp and concordant (epi)genotypes and discordant phenotypic presentations [37]. The sequence of events suggested by both Bliek et al. [37] and Boklage [36] are parallel and provide support that the cell populations containing the 11p15 imprinting anomaly are present extremely early during gestation and are likely present before the embryonic development stage.

The theory of diffused mosaicism was suggested by Cohen et al. [29] and has been accepted by others [38,39]. Complementary to this theory, we recently proposed the theory of epigenetic burden to suggest potential phenotypic consequence of diffused epigenetic mosaicism in the context of IC2 LOM and BWSp [26]. Application of this theory suggests that within the IC2 LOM population, the phenotypic presentation of patients with severe LOM could be considered similar to the spectrum of malformations associated with limb body wall complex, which could explain the later manifestation of malformations and anomalies throughout the fetal development period that may not be clinically visible until the surviving fetus is delivered [26]. Taken together, the phenotypes associated with cancer are likely a result of the 11p15 cell population present before or around the development of these systems. This supports the view that ‘prenatal and neonatal characteristic patterns’ represent a better ‘profile’ to understand the pleiotropic role that 11p15 provides in cancer predisposition. Here, we apply the underlying principles of these theories to hypothesize the role of 11p15 imprinting defects and cancer development in patients diagnosed with BWSp.

The lack of associations established between tumor development, patient sex or ART conception were surprising, as these contrast what would be expected from the general population epidemiology regarding WT and HB risk factors. A high rate of severe MZ-associated pregnancy findings was observed, suggesting this may represent a characteristic to explore in larger studies. Severe pregnancy complications such as twin-twin transfusion syndrome (TTTS) and twin anemia polycythemia sequence (TAPS) have been reported in some pregnancies with BWSp [37,40] in addition to vanishing twin [41]; however, it does not appear that a formal association has been established to date. We observed BWSp and cancer development associated with both donor and recipient roles in the transfusion, suggesting a complex dynamic within developing embryos. We observed a pregnancy complicated by twin reversed arterial perfusion (TRAP) sequence associated with a cardiac triplet and two surviving twins, which is exceptionally rare in the general population and the true incidence in triamniotic pregnancies is not known [42]. Further details regarding this unique family warrant separate publication. The 11p15 abnormal cell population was present prior to start of the unique developmental systems in all three fetuses. It appears that twinning without direct TTTS or severe complications may also provide an influence, as we observed multiple other patients with pregnancy histories that fit this ‘characteristic pattern’ within the case series.

From a pleiotropic perspective, one could hypothesize that genes within the 11p15 region may provide a mediating factor for the lack of common associations established with risk for tumor development within the population, as the BWSp phenotype (i.e., 11p15 anomalies) has been correlated to a variety of congenital anomalies. Some of these anomalies associated with BWS have also been associated with an increased HB risk and it has been suggested that individuals with BWS may represent some of the established HB risk factors [43]. A recent population-based study demonstrated that birth defects can mediate the relationship between childhood cancer and male sex predominance, with strong mediation effects identified specifically for hepatoblastoma and among children younger than one year at time of any cancer diagnosis [44].

The ‘Unified Theory of Evolution’ was proposed to describe the concepts that integrate environmental, epigenetic and genetic aspects to evolution [45]. In considering this integration within the BWSp, the sequence of events that lead to a patient presenting with BWSp features or a BWSp tumor can be organized into the following evolutional model to explore the role of abnormal 11p15 cell populations: (1) Peri-conception (parental demographic influences, fertility); (2) Conception (Natural or Assisted Reproduction); (3) Embryogenesis (Twinning, Developmental Axes, Patient Sex); (4) Embryonic Development (Limb-Body Wall, Craniofacial, Heart, Organs); (5) Fetal Development: (Anomalies/Malformations of Embryonic Stage); (6) Surviving Fetus which equals Birth.

The 11p15 cell population is likely present prior to the start of embryonic developmental systems. We found strong associations between the omphalocele and HI phenotypes and HB development; and organomegaly was demonstrated to be associated with tumor history within multiple different subpopulations. Specific to the pUPD11 population, it appears that craniofacial features may be associated with HB. Additional characteristics such as congenital anomalies of the kidney or urinary tract (CAKUT) and/or issues with adrenal glands were revealed as potential phenotypes associated with tumor development through the retrospective case series review, implicating abnormalities with enteric nervous system (ENS) development. The timing of abdominal wall and ENS development begins around the 3rd week of gestation. The resultant clinical features in BWSp suggest that the 11p15 imprinting abnormality cell population is present before this time period.

A significantly increased proportion of patients with HB and hyperinsulinism (HI) were observed in this study, which was an unexpected finding. As HI was only recently proposed as a cardinal feature by the BWSp-ICG, this relationship would not have been identified in previous cohorts. It has been suggested that significant overlap likely exists between mechanisms of tumorigenesis in liver and pancreas [46] supported pluripotent cells that contribute liver and pancreas tissues with possible trans-conversion between these two tissues [47]. Further research is needed to confirm whether HI does indeed influence cancer risk within BWSp patients as a variety of other tumor types was observed through our case series and in other reports [48,49,50]. We additionally identified a case report of a female infant with HI and NBL without features of BWS [51], representing a similar clinical ‘picture’ to the male patient (#21) we presented in the case series.

Although multiple tumor development was observed in some patients with severe BWSp phenotypes, we also observed multiple patients with severe BWSp phenotypes and no additional tumor development after more than 5–10 years of follow-up. These observations suggest that even in patients with history of cancer development, the complexities of diffused epigenetic cell burden cannot distinguish patients at risk for additional cancer development within this population. It appears that the 11p15 imprinting occurs extremely early after conception and that if not established properly within the first few days, can lead to asymmetric development and increased risk for cancer development. This may be due to a combination of diffused epigenetic mosaicism in addition to the burden of the epigenetic cells at particular key developmental junctions [36]. Evidence to support these findings also lies in the results of the discrepant 11p15 profiles established through clinical laboratory testing methods in our study and those reported by others [12,15,27]. Visible clinical evidence of these phenomena is available through the presenting phenotypes of individuals found to have mosaic 11p15 anomalies through testing.

The use of (epi)genotyping to predict cancer risk within the BWSp population was first suggested in 2004 [6] and this stratification concept has led to a current climate of unclear recommendations and international differences for cancer screening guidelines within this rare patient population. It has been accepted that mosaicism can present a challenge to molecular diagnostic confirmation of BWSp (11p15 anomaly) [27]. Our results demonstrate that in addition to mosaicism leading to positive or negative molecular testing in blood compared to tissues, some patients also have additional different mosaic epigenetic cell populations in tissues. Brzezinski et al. previously presented three patients with IC2 LOM and WT (or precursor) development, with one patient having IC1 GOM detected in some cell populations and clonality was suggested as a potential influence [12]. In the present study, we present five patients with IC2 LOM and HB development and three had discrepant profiles established between liver/HB and blood samples. In total, this now represents eight patients with mosaic IC2 LOM and tumor development, with three patients having possible mosaic IC1 GOM populations present within organ systems and one with mosaic IC2 LOM population present only in HB and not blood.

The tissue mosaicism observed in the BWSp-tumor population presented here suggests that the epigenotype profile established through clinical blood testing cannot necessarily be used to estimate or predict the 11p15-affected cell populations within an individual’s tissue and organ systems. Patient cohorts of classic BWS have demonstrated that the IC2 LOM profile is associated with lower cancer rates compared to patients with IC1 GOM or pUPD11 [2,7,8]. Our data demonstrating the full clinical spectrum of BWSp suggest that these established correlations within previous cohorts representing Classic BWS rather than the full BWS Spectrum may not be valid for the whole population of patients with an 11p15-associated anomaly (whether detectable in blood, or mosaic in tissues). Our results suggest that uncharacterized phenotypic features or other historical data variables may inform risk to a better extent than the BWSp phenotypic features designated by the ICG. Carli et al. recently proposed a prenatal clinical scoring system [41], which includes many of the features and characteristics classified within the prenatal ‘patterns’ identified in the present study; potential correlation between the prenatal system and tumor development should be explored in future investigations.

Tumor screening programs were proposed with the goal of a program to detect patients with an increased risk. The European medical system applies a 5% risk threshold for screening programs, while the US applies a more conservative >1% risk threshold approach [1,9,52]. In the present study, we observed tumor development rates approaching 5% risk for overall tumor development, with specific WT and HB rates approaching 2% risk within the IC2 LOM population, supporting the necessity to screen patients with this epigenotype profile established. We provide further data that suggest at least some patients with IC2 LOM profiled in blood samples may also have IC1 GOM cell populations present within the body, likely representing an influence for the tumor development–as these patients cannot be distinguished from patients with IC2 LOM without this additional mosaic GOM, it is not feasible to assume or ‘assign’ a risk of <2% to this subgroup.

While many patients with mosaic BWSp unfortunately present first with tumors before recognition of BWSp/11p15, the goal of a screening program is to identify patients at risk for tumors to determine and initiate the appropriate screening program based on personal risk (i.e., which tumors may develop and where to direct how to screen and what to ‘look for’). These screening programs and recommendations are augmented over time based on available evidence and expert group opinions, which led to the ICG-BWSp and AACR recommendations published independently in 2017 [9] and 2018 [1]. Although we recognize that tumor development in IC2 LOM is rare compared to pUPD11 and IC1 GOM, the emerging evidence from the literature and our cohort presented here suggest that tumor development may not be as rare as previously characterized, challenging the tumor screening stratification system applied to this group. The comparative rate of patients with IC2 LOM within the tumor development group in our BWSp Study Cohort (5/43) suggests that >10% of patients with BWSp and tumor development would not have received screening. We identified an additional four patients with IC2 LOM epigenotype profiles and HB tumor development through the BWSp Research Network Search, for a total of nine patients (*n* = 9) with IC2 LOM and tumors presented in this study. Anecdotally, we are aware of even more patients with IC2 LOM and tumor development without detailed data to suggest that our observations are not biased and likely represent emerging trends within the full BWSp population.

Blood samples may not provide an accurate representation of potential epigenotypes within an individual’s organ system populations, supporting the recommendations for universal screening in the BWSp population, rather than stratified by 11p15 (epi)genotype. In the interest of optimum patient care, we suggest that blood profiles may not be useful to stratify a patient’s individual tumor risk. Until additional data are clarified, universal tumor screening for a patient with clinical or molecular diagnosis of BWSp is warranted. The associations between LO and tumor development in patients with limited other common BWSp features suggests that the phenotype of ILO should be considered sufficient to warrant screening, even if the patient does not present with the ICG-BWSp clinical score of four points necessary for a clinical diagnosis. Of note, this population was also observed to have higher rates of tissue-mosaic 11p15 (with negative blood testing) through exploratory statistical analysis, further supporting that the blood 11p15 profile is not a useful tool for cancer prediction.

Additional controversy regarding specific methods for BWSp tumor screening also exist, with differing opinions regarding use of AFP screening for HB [1,9]. Our HB cohort was identified through AFP and ultrasound screening supporting the utility of AFP screening for HB in combination with full abdominal U/S as recommended by AACR and others [8,33]. We additionally observed excellent long-term outcomes within our case series and many patients with HB were diagnosed with low-stage tumors through screening that allowed for up-front resection.

There are several limitations of our study, which include potential cohort bias for the exploratory analyses, as previously discussed in the initial 2019 BWSp Cohort presentation [4]. Since this publication, multiple international cohorts have utilized these methods [19,20,21] providing strength in the methodology utilized in the present study and generalizability of our results. Furthermore, the BWS Registry is an international study and more than 10% of the cohort in this study are not from North America: 13 different countries/regions were represented in the present study cohort, including multiple countries in Europe and South America in addition to Australia and New Zealand. Within North America, patients from more than 47 states and Canada are enrolled in the BWS Registry. To prevent biased conclusions from the exploratory analyses, we additionally performed a selected case series review to provide further data to support our initial findings. The efforts behind this project involved more than two years of follow-up since the initial report to properly characterize patients by tumor development history and collection of detailed pregnancy histories, sample collection and molecular investigations through multiple collaborative research agreements and/or material transfer and data sharing agreements. These results highlight the utility and necessity of multi-institutional and international collaboration efforts to better understand populations of patients affected by rare diseases and/or cancer predisposition in order to advance personalized medicine stratifications by (epi)genotype.

## 5. Conclusions

In this study, we performed a large descriptive epidemiological investigation into the (epi)genotype, phenotype and population characteristics that could inform personal cancer risk within the BWSp population. Our results suggest that some phenotypes may be associated with tumor development; however, the heterogeneity of the BWSp population (both clinical and molecular) presents a challenge to understanding the full spectrum of BWSp. Within some population subgroups, we observed statistical associations between nephromegaly and cancer; and additionally identified a novel association between hyperinsulinism and hepatoblastoma. Mosaicism within both blood and tissue samples is frequent within individuals and the 11p15 anomalies can be present at different cell burden levels and/or as different epigenotype profiles within tissue populations compared to blood profiles. The tumor risk stratifications presented by the BWSp International Consensus group were derived from evidence within the classic BWS population and it is apparent from emerging evidence that these data are likely inaccurate for application to the full clinical BWSp population. Our results support the recommendations of the AACR for universal tumor screening for any patient suspected or confirmed of having an 11p15 anomaly. While most patients evaluated in this study were from North America, we did observe multiple international patients within our cohort, including one patient with IC2 LOM-WT from Europe and another with IC1 GOM-ART-WT from South America, suggesting these observations are not regional and likely extend to all patients with BWSp geographically. We advocate for reconsideration of universal tumor screening for BWSp within other countries and healthcare systems and further study of this complex BWSp population.

## Figures and Tables

**Table 1:** Frequency of expected and observed rates of cancer types that developed within BWSp study cohort population.

Characteristics and Subgroups		BWSp-ICG Data ^1^		BWSp Cohort Population	
**Study Cohort**	**Cancer/Tumor Type**	**Published Rates** **(%)**	**Expected** **(*n*)**	**Observed** **(*n*)**	**BWSp Rate** **(%)**
**BWSp Population Rate** (*n* = 215 individuals)	Overall Rate	~8%	17.2	43	20.0%
**Tumor Distributions** (*n* = 46 tumors)	Wilms Tumor (WT)	52% of all	22.4	29	63.0%
	Hepatoblastoma (HB)	14% of all	6.0	12	26.1%
	Neuroblastoma	10% of all ^2^	4.3	2	4.4%
	Other types	<5% of all	<2.2	3	6.5%
**BWSp (epi)Genotype Groups**	**Cancer/Tumor Type**	**Assigned Risk** **(%)**	**Expected** **(*n*)**	**Observed ** **(*n*)**	**Tumor Rate (%)**
**IC2 LOM Population** (*n* = 112 individuals)	Overall Rate	2.6%	2.9	5	4.5%
	Wilms Tumor (WT)	0.2%	0.2	2	1.8%
	Hepatoblastoma (HB)	0.7%	0.8	2	1.8%
	Neuroblastoma	0.5%	0.6	1	0.9%
**pUPD11 Population** (*n* = 73 individuals)	Overall Rate	16.0%	11.7	22	30.1%
	Wilms Tumor (WT)	7.9%	5.8	11	15.1%
	Hepatoblastoma (HB)	3.5%	2.6	9	12.3%
	Neuroblastoma	1.4%	1.0	1	1.4%
	Other types	~1%	0.8	1	1.4%
**IC1 GOM Population** (*n* = 30 individuals)	Overall Rate	28.1%	8.4	16	53.3%
	Wilms Tumor (WT)	24.0%	7.2	16	53.3%
	Other types	<2%	0.1	0	0%

^1^ Risk assigned by BWSp International Consensus Group (ICG) for each (epi)genotype groups; Data obtained from Table 3 and text (BWSp and embryonal tumors section, p. 17); Source: Brioude et al. 2018 [1]. ^2^ Tumor type most common to develop in patients with *CDKN1C* mutations (subtype not included within present study population).

**Table 2:** Ratio of males to females with and without tumor development in study population.

Tumor Type	Population Male:Female	Tumor Male:Female	No Tumor Male:Female
**Tumors (all)**			
Study Cohort (*n* = 215)	0.81 (96:119)	0.59 (16:27)	0.87 (80:92)
pUPD11 (*n* = 73)	0.83 (33:40)	0.69 (9:13)	0.89 (24:27)
IC2 LOM (*n* = 112)	0.84 (51:61)	0.67 (2:3)	0.85 (49:58)
**Wilms Tumor (WT)**			
Study Cohort (*n* = 201)	0.81 (90:111)	0.53 (10:19)	0.87 (80:92)
pUPD11 (*n* = 62)	0.88 (29:33)	0.83 (5:6)	0.89 (24:27)
IC1 GOM (*n* = 30)	0.67 (12:18)	0.45 (5:11)	1.00 (7:7)
IC2 LOM (*n* = 109)	0.82 (49:60)	0:2	0.85 (49:58)
**Hepatoblastoma (HB)**			
Study Cohort (*n* = 183)	0.87 (85:98)	0.83 (5:6)	0.87 (80:92)
pUPD11 (*n* = 60)	0.81 (27:33)	0.50 (3:6)	0.89 (24:27)
IC2 LOM (*n* = 109)	0.88 (51:58)	2:0	0.85 (49:58)

**Table 3:** Rate of tumor development among ART conception and multiple gestation pregnancies.

Population Group	Total (*n*)	No Tumor (*n*)	Tumor (*n*)	Tumor Rate %	Tumor Types
BWSp Population	215	172	43	20.0%	29 WT, 11 HB, 2 NBL, 3 other
**ART Conception ^1^**					**Tumor Types**
IC2 LOM	34	32	2	5.9%	1 HB, 1 WT
pUPD11	3	3	0	0%	-
IC1 GOM	3	1	2	66.7%	2 WT
BWSp Cohort	40	36	4	10.0%	3 WT, 1 HB
**Multiple Gestation**					**Tumor Types**
IC2 LOM	22	19	3	13.6%	1 HB *, 1 WT *, 1 NBL
pUPD11	6	5	1	16.7%	1 HB ^1^
IC1 GOM	3	2	1	33.3%	1 WT *
BWSp Cohort	31	26	5	16.1%	2 WT, 2 HB, 1 NBL
Tumors in Multiple Gestation by Conception	31	26	5	16.1%	ART ^2^: 2 WT, 1 HB NAT: 1 HB, 1 NBL

Tumor Type Abbreviations: HB = Hepatoblastoma; NBL = Neuroblastoma; WT = Wilms Tumor. ^1^ Patient developed adrenal mass prior to HB development. ^2^ ART = Assisted Reproductive Techniques utilized for conception; Conception types included IVF and/or ICSI (*n* = 38) and ‘other ART’ (*n* = 2 IC1 GOM). * Tumors associated with both ART conception and multiple gestation in patient.

**Table 4:** Summary of phenotypes associated with cancer history in BWSp study population.

Phenotype Feature	Characteristic	Total (*n* = 215)	No Tumor (*n* = 172)	Tumor (*n* = 43)	*p*-Value
**Conception Type**	Natural	80.0%	77.4%	90.2%	0.066 ^1^
	IVF/ICSI	19.0%	22.0% ^1^	7.3% ^1^	
	Other ART	14.8%	0.6%	2.4%	
**Prenatal and Birth**	Multiple gestation	15.1%	16.0%	11.9%	0.633
	ICG-Pregnancy	34.4%	36.4%	26.3%	0.259
	Preterm Birth	40.9%	40.5%	42.5%	0.859
	Large for GA	63.5%	63.6%	63.2%	1.000
**Common BWSp Features**	Macroglossia	71.8%	77.8%	47.6%	<0.001 ***
	BWSp-LO	73.8%	72.0%	81.0%	0.325
	Facial Nevus Simplex	51.0%	58.5%	20.5%	<0.001 ***
	Ear creases/pits	62.2%	67.1%	42.1%	0.005 **
**Hypoglycemia**	**Overall Rate**	60.1%	64.2%	43.9%	0.021 *
	Severe (HI)	20.9%	21.4%	18.6%	0.834
	Transient	38.4%	42.0%	24.4%	0.048 *
**Abdominal Wall Defects (AWD)**	**Overall Rate**	70.1%	75.0%	50.0%	0.003 **
	Omphalocele	24.1%	26.6%	14.0%	0.109
	Minor Defect	45.1%	47.6%	35.0%	0.162
**Organomegaly**	**Overall Rate**	33.7%	31.6%	41.5%	0.267
	Nephromegaly	19.6%	16.8%	30.0%	0.074
	Hepatomegaly	19.8%	19.6%	20.5%	1.000
	Splenomegaly	14.5%	13.6%	17.9%	0.456
**Source of BWSp Diagnosis**	Blood +	82.5%	87.2%	64.3%	0.001 **

^1^ Difference by column proportion testing. * = *p* < 0.05; ** *p* < 0.01; *** *p* < 0.001.

**Table 5:** Tumor phenotype associations within BWS spectrum population groups.

	**Characteristic Present**	**No Tumor** **(*n* = 124)**	**Tumor** **(*n* = 20)**	***p*-Value**
		% (observed)	% (observed)	
**Classic BWS Phenotype ** **Population (*n* = 144)**	Molecular Testing (+ Blood)	97.4% (113)	100% (20)	1.000
	Preterm Birth	47.5% (57)	80.0% (16)	0.008 **
	Macroglossia	93.5% (116)	95.0% (19)	1.000
	BWSp-LO	72.3% (86)	75.0% (15)	1.000
	Abdominal Wall Defect	87.5% (105)	75.0% (15)	0.166
	Hypoglycemia (any)	68.3% (82)	70.0% (14)	1.000
	Facial Nevus Simplex (FNS)	69.2% (81)	38.9% (7)	0.017 *
	Organomegaly	38.3% (44)	65.0% (13)	0.030 *
	Nephromegaly	21.1% (23)	52.6% (10)	0.008 **
	Hepatomegaly	24.1% (26)	33.3% (6)	0.394
	**Characteristic Present**	**No Tumor** **(*n* = 40)**	**Tumor** **(*n* = 22)**	***p*-Value**
**Atypical BWSp/** **BWSp-ILO Phenotypes Population (*n* = 62)**	Molecular Testing (+ Blood)	55.0% (22)	28.6% (6)	0.062 ^1^
	Preterm Birth	23.1% (9)	5.0% (1)	0.141
	Macroglossia	27.5% (11)	4.5% (1)	0.042 *
	BWSp-LO	72.5% (29)	85.7% (18)	0.342
	Abdominal Wall Defect	37.5% (15)	25.0% (5)	0.395
	Hypoglycemia (any)	51.3% (20)	19.0% (4)	0.026 *
	Severe (HI)	35.0% (14)	4.5% (1)	0.011*
	Transient	15.4% (6)	14.3% (3)	1.000
	Organomegaly	13.2% (5)	9.5% (2)	0.708
	Nephromegaly	5.3% (2)	9.5% (2)	0.611
	Hepatomegaly	8.0% (3)	9.5% (2)	1.000

^1^ Difference by column proportion testing. * = *p* < 0.05; ** *p* < 0.01.

**Table 6:** Tumor phenotype associations within lateralized overgrowth (BWSp-LO) population.

Lateralized Overgrowth (LO) Population Phenotypes	BWSp-LO Group Total (*n* = 152)	BWSp-LO No Tumor (*n* = 118)	BWSp-LO Tumor (*n* = 34)	*p*-Value
**BWSp Subtypes**				<0.001 ***
IC1 GOM	13.8% (21)	8.5% (10)	32.4% (11)	
pUPD11	45.4% (69)	40.7% (48)	61.8% (21)	
IC2 LOM	40.8% (62)	50.8% (60)	5.9% (2)	
**Molecular Testing (+ Blood)**	77.7% (115)	84.2% (96)	55.9% (19)	0.002 **
**Macroglossia**	64.9% (98)	71.2% (84)	42.4% (14)	0.004 **
**Facial Nevus Simplex**	47.6% (68)	55.0% (61)	21.9% (7)	0.001 **
**AWD (any)**	63.9% (92)	70.5% (79)	40.6% (13)	0.003 **
Omphalocele	18.0% (27)	19.0% (22)	15.6% (5)	0.800
Minor defect	45.1% (65)	50.9% (57)	25.0% (8)	0.015 *
**Hypoglycemia (any)**	63.4% (92)	69.0% (78)	43.8% (14)	0.012 *
Severe (HI)				0.819
Transient	38.6% (56)	43.4% (49)	21.9% (7)	0.039 *

+ = blood positive. * = *p* < 0.05; ** *p* < 0.01; *** *p* < 0.001.

**Table 7:** Significant tumor phenotype associations within BWSp—Organomegaly population.

Organomegaly Population Phenotypes	Organomegaly Group Total	Organomegaly No Tumor	Organomegaly Tumor	*p*-Value
	**(*n* = 66)**	**(*n* = 49)**	**(*n* = 17)**	
**Male sex**	53.0% (35)	63.3% (31)	23.5% (4)	0.010 *
**Diversity groups**				0.043 *
‘White/Caucasian’ only	63.5% (40)	71.7% (33)	41.2% (7)	^1^
‘Mixed’ race/ethnicity	17.5% (11)	10.9% (5)	35.3% (6)	^1^
‘Other’ race/ethnicity	19.0% (12)	17.4% (8)	23.5% (4)	
**Specific Organs Affected**				
Nephromegaly	62.7% (37)	58.1% (25)	75.0% (12)	0.365
Hepatomegaly	64.9% (37)	69.1% (29)	53.3% (8)	0.349
Splenomegaly	33.9% (19)	36.6% (15)	26.7% (4)	0.543

^1^ Difference by column proportion testing. * = *p* < 0.05.

**Table 8:** Wilms (WT)-associated phenotypes within BWSp population.

Characteristic	No Tumor (*n* = 172)	BWSp-WT (*n* = 29)	*p*-Value
**Blood +**	87.2% (143)	53.6% (15)	<0.001 ***
**Conception Type**			0.081 ^1^
Natural	77.4% (123)	89.3% (25)	
IVF/ICSI	22.0% (35)	7.1% (2)	
Other ART	0.6% (1)	3.6% (1)	
**BWSp Features**			
Macroglossia	77.8% (133)	34.5% (10)	<0.001 ***
BWSp-LO	72.0% (118)	75.0% (21)	0.823
Ear creases/pits	67.1% (104)	26.9% (7)	<0.001 ***
Facial Nevus Simplex	58.5% (93)	14.8% (4)	<0.001 ***
**Organomegaly**	31.6% (49)	26.9% (10)	0.666
Nephromegaly	16.8% (25)	25.9% (7)	0.280
Hepatomegaly	19.6% (29)	22.2% (6)	0.795
**Hypoglycemia (any)**	64.2% (104)	35.7% (10)	0.006 **
Severe (HI)	21.4% (36)	3.4% (1)	0.020 *
Transient	42.0% (68)	32.1% (9)	0.406
**AWD (any)**	75.0% (123)	33.3% (9)	<0.001 ***
Omphalocele	26.6% (45)	0/29	<0.001 ***
Minor Defect	47.6% (78)	33.3% (9)	0.212

^1^ Difference by column proportion testing. * = *p* < 0.05; ** *p* < 0.01; *** *p* < 0.001. + + blood positive.

**Table 9:** Hepatoblastoma (HB)-associated phenotypes within BWSp population.

Characteristic	No Tumor (*n* = 172)	BWSp-HB (*n* = 11)	*p*-Value
**Blood +**	87.2% (143)	100% (11)	0.365
**Preterm Birth**	40.5% (66)	80.0% (8)	0.020 *
**Large Size (LGA)**	63.6% (105)	88.9% (8)	0.163
**BWSp Features**			
Macroglossia	77.8% (133)	90.0% (9)	0.692
BWSp-LO	72.0% (118)	100% (11)	0.069 ^1^
Ear creases/pits	67.1% (104)	100% (9)	0.058 ^1^
**Organomegaly**	31.6% (49)	60.0% (6)	0.085
Nephromegaly	16.8% (25)	40.0% (4)	0.085
Hepatomegaly	19.6% (29)	22.2% (2)	1.000
**Hypoglycemia (any)**	64.2% (104)	70.0% (7)	1.000
Severe (HI)	21.4% (36)	54.5% (6)	0.022 *
Transient	42.0% (68)	10.0% (1)	0.052 ^1^
**Abdominal Wall Defect**	75.0% (123)	90.0% (9)	0.454
Omphalocele	26.6% (45)	54.5% (5)	0.182
Minor Defect	47.6% (78)	40.0% (4)	0.751

^1^ Difference by column proportion testing. * = *p* < 0.05. + = blood positive.

**Table 10:** Wilms tumor (WT)-associated phenotypes within pUPD11 population.

pUPD11 Characteristics	pUPD11 No Tumor (*n* = 51)	pUPD11-WT (*n* = 11)	*p*-Value
**Blood +**	63.3% (31)	36.4% (4)	0.174
**Preterm Birth**	22.9% (11)	10.0% (1)	0.670
**BWSp Features**			
Macroglossia	40.0% (20)	18.2% (2)	0.299
BWSp-LO	96.0% (48)	90.9% (10)	0.455
Facial Nevus Simplex	35.4% (17)	0/11	0.024 *
**Hypoglycemia (any)**	81.6% (40)	30.0% (3)	0.002 **
Severe (HI)	42.9% (21)	9.1% (1)	0.043 *
Transient	38.8% (19)	20.0% (2)	0.470
**Organomegaly**	28.3% (13)	27.3% (3)	1.000
Nephromegaly	13.3% (6)	27.3% (3)	0.358
Hepatomegaly	24.4% (11)	18.2% (2)	1.000
Splenomegaly	13.3% (6)	36.4% (4)	0.093
**Abdominal Wall Defect**	57.1% (28)	18.2% (2)	0.042 *
Omphalocele	4.2% (2)	0/11	1.000
Minor defect	53.1% (26)	18.2% (2)	0.048 *

* = *p* < 0.05; ** *p* < 0.01. + = blood positive.

**Table 11:** Hepatoblastoma (HB)-associated phenotypes within pUPD11 population.

pUPD11 Characteristics	pUPD11 No Tumor (*n* = 51)	pUPD11-HB (*n* = 9)	*p*-Value
**Blood +**	63.3% (31)	100% (9)	0.045 *
**Preterm Birth**	22.9% (11)	75.0% (6)	0.007 **
**BWSp Features**			
Macroglossia	40.0% (20)	87.5% (7)	0.020 *
BWSp-LO	96.0% (48)	100% (9)	1.000
Ear creases/pits	46.8% (22)	100% (7/7)	0.012 *
**Hypoglycemia (any)**	81.6% (40)	62.5% (5)	0.345
Severe (HI)	42.9% (21)	55.6% (5)	0.717
Transient	38.8% (19)	0/8	0.042 *
**Organomegaly**	28.3% (13)	75.0% (6)	0.017 *
Nephromegaly	13.3% (6)	50.0% (4)	0.033 *
Hepatomegaly	24.4% (11)	28.6% (2)	1.000
**Abdominal Wall Defect**	57.1% (28)	87.5% (7)	0.134
Omphalocele	4.2% (2)	33.3% (3)	0.024 *
Minor defect	53.1% (26)	50.0% (4)	1.000

* = *p* < 0.05; ** *p* < 0.01. + = blood positive.

**Table 12:** Epigenotype profiles (blood) and phenotype patterns associated with tumor development in BWSp.

Blood Profile Established	Patient (Sex)	11p15 Epigenotype Profile			BWSp Phenotype and Tumor Profiles		
		IC2 %	IC1 %	Affected Cell Fraction	Phe-Score and Group	Tumor Type	BWSp Dx + Screening
IC2 LOM (Normal IC1)	#01 (Male) ^1^	0.05% (+)	48.16% (N)	99.90%	14 (Severe)	HB	+
	#02 (Male)	0.99% (+)	53.70% (N)	98.02%	10 (Severe)	HB (recur)	+
	#03 (Female) ^1^	23.75% (+)	49.42% (N)	52.50%	8 (Classic)	NBL	+
	#04 (Female)	27.12% (+)	50.41% (N)	45.76%	6 (Atypical)	HB	+
	#05 (Female)	33.89% (+)	49.60% (N)	32.22%	1 (UH)	WT	-
	#06 (Male) ^1^	40.44% (+)	(N)	19.12%	No Features	HB	-
IC2 LOM + IC1 GOM (pUPD11)	#07 (Female)	13.06% (+)	80.39% (+)	67.33%	11 (Severe)	HB (+PBL)	+
	#08 (Female)	16.87% (+)	80.27% (+)	63.40%	11 (Severe)	HB (x2)	+
	#09 (Male)	33.52% (+)	72.66% (+)	37.14%	11 (Severe)	HB	+
	#10 (Male)	36.99% (+)	61.33% (+)	24.34%	9 (Classic)	HB	+
	#11 (Female)	41.03% (+)	59.74% (+)	18.71%	LO	HB	Adult Dx
	#12 (Female)	46.04% (+)	58.22% (+)	12.18%	9 (Classic)	HB	C
IC1 GOM (Normal IC2)	#13 (Female)	49.60% (N)	68.97% (+)	37.94%	8 (Classic)	Bilat Nephr	+
	#14 (Female) ^1^	(N)	56.28% (+)	12.56%	None (+IVF)	WT	-
	#15 (Female) ^1^	(N)	56.23% (+)	12.46%	LO	HB	-
Normal Methylation	#16 (Female)	49.96% (N)	48.10% (N)	<3% (N)	4 (Atypical/ILO)	HB	-
	#17 (Male) ^1^	48.52% (N)	52.33% (N)	<3% (N)	4 (Atypical/ILO)	WT	-

^1^ Previous reports of epigenotype data in patients include: #01 (ID#03 in [26]); #03 (ID#26 in [26]); #06 (No. 23 in [15]); #14 (No. 3 in [15]); and #15 (No. 22 in [15]). Blood testing results for patient #17 new since original description (Patient 9 in [27]). Table Abbreviations: (+) = abnormal methylation in region (GOM in IC1 or LOM in IC2); (N) = normal methylation; Phe-Score = BWSp clinical score and phenotype group, UH = umbilical hernia; BWSp Dx + Screening = BWSp diagnosis prior to detection of tumor, C = concordant diagnoses); Bilat Nephr = Bilateral nephroblastomatosis.

**Table 13:** 11p15 Epigenotype profiles associated with hepatoblastoma (HB) and IC2 LOM.

Patient ID	Sample Type	BWSp Epigenotype Profile Established				
		IC2 %	IC1 %	11p15 Copy Number ^1^	11p15 DMR Influence	Affected Cell Fraction
Patient #01 (Male)	Blood	0.05% (+)	48.16% (N)	(N)	IC2	99.90%
	Liver (normal)	2.70% (+)	50.24% (N)	-	IC2	94.60%
	HB Tumor	0.91% (+)	47.86% (N)	-	IC2	98.18%
	Placenta	30.32% (+)	47.05% (N)	-	IC2	39.36%
Mother	Saliva	48.88% (N)	47.90% (N)	-	No 11p15	<3%
Patient #04 (Female)	Blood	27.12% (+)	50.41% (N)	(N)	IC2	45.76%
	Liver (normal)	24.00% (+)	62.02% (+)	(N)	IC2 + IC1	38.02%
	HB Tumor	13.65% (+)	57.84% (+)	(N)	IC2 + IC1	44.19%
Patient #18 (Female)	Blood	Hypo	(N)	(N)	IC2	Cannot Assess ^2^
	Liver (normal)	15.67%	51.81%	-	IC2	68.66%
	HB Tumor	25.13%	52.19%	-	IC2	49.74%
Patient #16 (Female)	Blood	49.96% (N)	48.10% (N)	(N)	No 11p15	<3%
	HB Tumor	13.32% (+)	51.32% (N)	(N)	IC2	49.74%
Patient #06 (Male)	Blood	40.44% (+)	(N)	(N)	IC2	19.12%
	HB Tumor	7.12% (+)	80.42% (+)	(N)	IC2 + IC1	73.30%
Familial Testing	Mother	36.81% (+)	(N)	(N)	IC2	26.38%
	Mat GDM	(N)	(N)	-	No 11p15	<3%
	Mat GDF	(N)	(N)	-	No 11p15	<3%

^1^ Copy number of 11p15 assessed by SNP array and/or high density aCGH array. (+) = positive, (N) = Normal. ^2^ Testing performed at clinical laboratory that reported ‘normal methylation’ or ‘hyper/hypo’ without specific data values.

## Data Availability

The data presented in this study are available in the article, Supplementary Materials Tables S1–S5, Supplementary Appendix A: Contains list of abbreviations, data variables and additional methodology for the BWS Reg-istry database and specific methods utilized in this study, Supplementary Tables A1–A3, Exploratory Analyses Methodology, Case Series Review Methodology (section); Supplementary Appendix B: Supplementary Tables B1–B17, Contains all supporting tables and data for results.

## Supplementary Materials

The following are available online at https://www.mdpi.com/article/10.3390/genes12111839/s1, Supplementary Appendix A: Detailed Methodology for BWSp Study Cohort and Selected Case Series. Supplementary Appendix B: Supporting Data and Information for Study Results. Table S1: Characteristics Associated with Multiple Tumor Development in Patients with BWSp due to pUPD11, Table S2: Epigenotype and Somatic HB Profiles Established in Samples Collected from Patients with pUPD11 and Multiple Tumor Development, Table S3: Comparison of Clinical Characteristics and Hepatoblastoma (HB) Development History in Two Male Patients with IC2 LOM, Table S4: Additional Evidence for Tissue Mosaicism Associated with ART, IC1 GOM and WT Development, Table S5. Evidence for Tissue Mosaicism Associated with Neuroblastoma (NB) Development in Two Patients with BWSp and Epigenotypes other than CDKN1C mutations.
