# Supplementary material for: Characteristics Associated with Tumor Development in Individuals Diagnosed with Beckwith–Wiedemann Spectrum: Novel Tumor-(epi)Genotype-Phenotype Associations in the BWSp Population
Source: Genes (Basel). 2021 Nov 21;12(11):1839. doi: 10.3390/genes12111839 (PMC8621885; doi:10.3390/genes12111839)
Supplement: Supplementary file 1 [file genes-12-01839-s001.zip › genes-1465311-supplementary.pdf]

APPENDIX A: Detailed Methodology for BWSp Study Cohort and Selected Case Series

CONTENT SUMMARY:

List of Common Abbreviations (Table A1) page: 2

Description of BWS Registry Database Variables (Table A2) pages: 3-4

Exploratory Analyses Methodology (section) pages: 5-7

Case Series Review Methodology (section) page: 8
Includes BWSp Scoring Methods (Table A3)

Table A1. List of Abbreviations Commonly Used in Study Data Tables.

Abbreviation	Definition
11p15	Chromosome 11 region associated with BWSp
AACR	American Association for Cancer Research
AFP	Alpha-fetoprotein
ART	Assisted Reproductive Techniques
AWD	Abdominal Wall Defect
BWS	Beckwith-Wiedemann Syndrome
BWSp	Beckwith-Wiedemann Spectrum
BWSp-ICG	International Consensus Group for BWSp
DMR	Differentially methylation region
FNS	Facial Nevus Simplex
GI	Gastrointestinal
GOM	Gain of Methylation (hypermethylation)
GU	Genitourinary
HB	Hepatoblastoma
HI	Hyperinsulinism
ILO	Isolated Lateralized Overgrowth (or asymmetry)
IC1	Imprinting Control region 1 (*H19*/*IGF2*:IG-DMR)
IC2	Imprinting Control region 2 (*KCNQ1OT1*:TSS-DMR)
ICG	International Consensus Group
ICG-Pregnancy	Suggestive pregnancy features included in BWSp-ICG clinical scoring system (polyhydramnios and/or placentomegaly)
ICSI	Intracytoplasmic sperm injection
IVF	In Vitro Fertilization
LGA	Large for gestational age (>2 standard deviations above mean)
LO	Lateralized Overgrowth (or asymmetry)
LOM	Loss of Methylation (hypomethylation)
NBL	Neuroblastoma
PBL	Pancreatoblastoma
pUPD11	Paternal uniparental isodisomy of chromosome 11
SNP array	Single nucleotide polymorphism (SNP) array
TRAP	Twin Reversed Arterial Perfusion (TRAP) Sequence
TTTS	Twin-Twin Transfusion Syndrome
WT	Wilms Tumor

Table A2. Description and Definition of Variables Selected from the 2019 BWSp Registry database.

Variable Category and Subgroups	Definition, Criteria, and/or Notes
**BWSp Epigenotype Groups**	BWSp subtype identified in patient sample with positive 11p15 abnormality (i.e. blood or other tissue if blood negative or not available)<br>Blood utilized as 'reference subtype' (positive or negative) with tissue considered secondary source
IC1 GOM	DMR1 involvement only
IC2 LOM	DMR2 involvement only
pUPD11	DMR1 and DMR2 involvement
**BWSp Spectrum Groups**	**Criteria established for 2019 BWSp cohort published*****
Classic BWS/BWSp	Clinical score ≥6 points with ≥2 cardinal features<br>(except if 2 cardinal features present=HI + LO)
Atypical BWSp	Clinical score <6 points with ≥1 cardinal feature; OR<br>Clinical score ≥6 points with HI+LO as only 2 cardinal features; OR<br>Presenting feature for BWSp diagnosis was HI and/or Tumor
Isolated LO	Clinical score <4 with LO as only cardinal feature
**Positive (+)Blood Testing**	Positive 11p15 methylation abnormality in blood sample<br>*Patients with negative blood testing were found to have positive 11p15 in at least one tissue sample*
**Patient sex**	Male or Female *(male sex selected as reference)*
**Diversity Groups**	**Self-reported demographics**
'White/Caucasian' only	White or Caucasian and non-Hispanic
'Mixed' race/ethnicity	More than one race reported, or race with Hispanic ethnicity reported
'Other' race/ethnicity	Diversity group other than white, or identified as Hispanic only
**Conception Type**	
Natural	Spontaneous conception without any form of ART utilized
IVF/ICSI	IVF and/or ICSI reported or documented as fertility method
Other ART	Other form of ART reported or documented<br>Includes hormone stimulation, intrauterine insemination (IUI), etc
**Multiple Gestation**	Patient classified as twin or higher order (or singleton) at birth
**ICG-Pregnancy**	Features during pregnancy classified as 'suggestive' in scoring system
Polyhydramnios	Increased fluid levels (subjectively noted or requiring amnioreduction)
Placentomegaly	Large placenta for GA (subjectively noted or through pathology)
**Preterm Birth**	Gestational Age (GA) less than 37 weeks<br>Criteria defined by World Health Organization (WHO)
**Common Features**	
Macroglossia	Tongue enlargement; Includes asymmetric enlargement
BWSp-LO	Lateralized Overgrowth due to BWSp/11p15<br>Defined as visible muscle bulk difference or >5% measurable size discrepancy
Ear creases/pits	Linear indents/grooves on earlobes or small holes near helix
Facial Nevus Simplex	Reddish appearance on forehead, eyelids, and/or nose (red birthmark on face that resolves – not port-wine stain)

Large Size (LGA)	Birthweight classified as Large for Gestational Age (>2SDs above the population mean) or 'LGA at birth' noted on birth records
**Abdominal Wall Defects**	Malformation of abdominal wall development<br>*(can be Severe, Mild, or Not Present in patients with BWSp)*
Omphalocele	Severe form of AWD
Minor defect	Umbilical hernia and/or diastasis recti<br>Individuals with history of omphalocele recorded as 'no minor defect'
**Hypoglycemia**	
Severe (HI)	Hyperinsulinism lasting >1 week and requiring escalated treatment
Transient	Hypoglycemia lasting <1 week
**Organomegaly**	**Enlarged organ or organs, including spleen; Recorded for patients with available data pre-tumor diagnosis**
Nephromegaly	Kidney enlargement (includes asymmetric enlargement of one kidney or bilateral enlargement)
Hepatomegaly	Liver enlargement on imaging or by palpation (hepatosplenomegaly)
Splenomegaly	Spleen enlargement on imaging or by palpation (hepatosplenomegaly)

EXPLORATORY ANALYSES METHODOLOGY SECTION

Description of comparison group selection and case-control criteria applied for each population:

BWSp Study Population

Inclusions: all patients in study cohort (n=215); Subtypes: IC2 LOM, IC1 GOM, pUPD11
Exclusions Applied: none
Case Criteria: History of Tumor Development (n=43)
Control Criteria: No history of tumor development (n=172)
Full Results Available: Appendix B: Table B3

Classic BWS Phenotype Population

Inclusions: patients classified with 'Classic BWSp' in 2019 cohort (n=144)
Exclusions Applied: patients classified with 'Atypical BWSp' or 'ILO' (or those unclassified)
Case Criteria: Tumor - Classic BWS Phenotype (n=20)
Control Criteria: No Tumor – Classic BWS Phenotype (n=124)
Full Results Available: Appendix B: Table B4

Atypical BWSp/ ILO Phenotype Population

Inclusions: patients classified with 'Atypical BWSp' or 'ILO' phenotypes in 2019 cohort (n=62)
Exclusions Applied: patients classified with 'Classic BWSp' (or those unclassified)
Case Criteria: Tumor – Atypical BWSp/ILO Phenotype (n=22)
Control Criteria: No Tumor – Atypical BWSp/ILO Phenotype (n=40)
Full Results Available: Appendix B: Table B5

Lateralized Overgrowth (LO) Phenotype Population

Inclusions: patients with LO recorded as clinical feature (n=152)
Exclusions Applied: patients with LO recorded as 'not present/affected' (or those with LO status unknown)
Case Criteria: Tumor – LO Phenotype (n=34)
Control Criteria: No Tumor – LO Phenotype (n=118)
Full Results Available: Appendix B: Table B6

Organomegaly Phenotype Population

Inclusions: patients with organomegaly recorded as clinical feature (n=66); includes enlargement of kidneys, liver, and/or spleen
Exclusions Applied: patients with organomegaly recorded as 'not present' (or those with organomegaly status unknown)
Case Criteria: Tumor – Organomegaly Phenotype (n=17)
Control Criteria: No Tumor – Organomegaly Phenotype (n=49)
Full Results Available: Appendix B: Table B7

Wilms Tumor (WT)-Phenotype Comparisons

Inclusions: study cohort with history of WT development and those without history of any tumor development (n=201)
Exclusions Applied: patients with history of development of HB or other tumor type
Case Criteria: patients with WT/bilateral nephroblastomatosis development (BWSp-WT, n=29)
Control Criteria: No Tumor Population (n=172)
Full Results Available: Appendix B: Table B8

Hepatoblastoma (HB)-Phenotype Comparisons

Inclusions: study cohort with history of HB development and those without history of tumor development (n=183)
Exclusions Applied: patients with history of development of WT or other tumor type
Case Criteria: patients with HB development; (BWSp-HB, n=11)
Control Criteria: No Tumor Population (n=172)

Note – same 'control group' for WT and Tumor-Phenotype comparisons

Full Results Available: Appendix B: Table B9

IC1 GOM-BWSp Population = all patients with IC1 GOM in study cohort (n=30)

Exclusions Applied: patients with IC2 LOM or pUPD11
Case Criteria: History of Tumor Development (n=16)
Control Criteria: No history of tumor development (n=14)
Full Results Available: Appendix B: Table B10

IC2 LOM-BWSp Population = all patients with IC2 LOM in study cohort (n=112)

Exclusions Applied: patients with IC1 GOM or pUPD11
Case Criteria: History of Tumor Development (n=5)
Control Criteria: No history of tumor development (n=107)
Full Results Available: Appendix B: Table B11

pUPD11-BWSp Population = all patients with pUPD11 in study cohort (n=73)

Inclusion: patients with both IC1 GOM and IC2 LOM due to pUPD11
Exclusions Applied: patients with IC2 LOM or IC1 GOM only
Case Criteria: History of Tumor Development (n=22)
Control Criteria: No history of tumor development (n=51)
Full Results Available: Appendix B: Table B12

pUPD11-WT Phenotype Comparisons

Inclusions: patients in study cohort with pUPD11 and history of WT development, and those with pUPD11 without history of tumor development (n=62)
Exclusions Applied: patients with pUPD11 and history of development of HB and/or other tumor
Case Criteria: pUPD11-WT Population (n=11)
Control Criteria: pUPD11 No-Tumor Population (n=51)
Full Results Available: Appendix B: Table B13

pUPD11-HB Phenotype Comparisons

Inclusions: patients in study cohort with pUPD11 and history of HB development, and those with pUPD11 without history of tumor development (n=60)

Exclusions Applied: patients with pUPD11 and history of development of WT or other tumor type

Case Criteria: pUPD11-HB Population (n=9)

Control Criteria: pUPD11 No-Tumor Population (n=51)

Note – same 'control group' for WT and Tumor-Phenotype comparisons

Full Results Available: Appendix B: Table B14

CASE SERIES REVIEW METHODOLOGY SECTION

Additional Characteristics of Interest in Record Review:

To evaluate additional characteristics and phenotype data collected during the retrospective case series review, we designated the following variable groups:

- Peri-Conception Profile: (1) Type of conception; (2) singleton/multiple; (3) history of twinning or infertility in immediate family members (siblings, parents).
- Prenatal Environment Profile: (1) Pregnancy complications; (2) Prenatal phenotypes detected through imaging.
- Neonatal Profile: (1) Birth outcome; (2) Phenotype presentation during neonatal period (<30 days); (3) Historical prenatal phenotype (noted subjectively or detected through imaging).
- BWSp Profiles: (1) ICG Clinical Score; (2) BWS Spectrum Group; (3) Epigenotype profile(s) established in designated clinical testing laboratories; (4) Results of other laboratories (as applicable).
- Tumor Presentation Profile: (1) Type of tumor(s) that developed; (2) Detection type: BWSp screening, Incidental (other screening), Symptomatic; (3) Tumor detected pre- or post-BWSp diagnosis.

Methodology for BWSp Clinical Scoring System:

Table A3. Methodology for BWSp Clinical Scoring System.

Cardinal Features (2 points each)	Macroglossia; Omphalocele; LO; HI; Multifocal/bilateral WT or nephroblastomatosis; Pathology Features (adrenal cortex cytomegaly, placental mesenchymal dysplasia, or pancreatic adenomatosis)
Suggestive Features (1 point each)	Pregnancy; LGA; FNS; ear creases/pits; minor AWD; transient hypoglycemia; nephromegaly/hepatomegaly; Typical BWSp tumor
ICG Score System	(# Cardinal Features x 2 points) **+** (# Suggestive Features x 1 point)
Study Score System	Tumors not included in scoring; Pathology included for scores in present study *(was not included in 2019 study scores)*

APPENDIX B: Supporting Data and Information for Study Results

Additional Population Characteristics (Tables B1 and B2) **pages: 2 - 3**

Exploratory Analyses Results (Tables B3 – B14) **pages: 4 - 15**

Univariate comparisons were performed to evaluate the frequency of characteristics/features between the case-control groups to evaluate whether significant associations or trends were present between groups that could inform tumor-phenotype profiles. Categorical variables were compared with Pearson chi-square, and nominal variables were compared using Fisher's Exact; column proportion (z-testing) with Bonferroni correction methods was performed for all comparisons. Statistical significance was set at p<0.05 for all comparisons. The significance of the associations identified for each variable are differentiated by asterics for the strength of p-value demonstrated: *=p<0.05; **p<0.01; ***p<0.001 (as appropriate). Significant differences identified through column proportion testing are denoted with footnotes in each of the tables. All statistical analyses were performed using IBM SPSS Statistics Version 26.

Case Series Supporting Data (Tables B15 – B17) **pages: 16 - 18**

Table B1. Length of follow-up and confirmation of tumor development status in cohort.

	Tumor Development Status Confirmed [1]			
	Dataset Review		Follow-Up	
**Review Period**	March 2019	December 2020	June 2021 [4]	
**Total Patients Followed**	215	215	215	
**Total Years of Follow-Up** [2]	1058 years	1426 years	1481 years	
**Average per patient**	4.9 years	6.6 years	6.9 years	
**Median per patient**	3y [IQR 1y;5y]	5y [IQR 3y;7y]	5y [IQR 3y;7y]	
**Age at Last Follow-Up**	**Reviewed (n=215)**	**Reviewed (n=215)**	**No Additional Review (n=99)**	**Detailed Review (n=116)**
Birth – 1.9 years	72	8	7	-
2 years – 3.9 years	59	43	17	17
4 years – 6.9 years	37	101	49	40
7 years – 9.9 years	18	19	5	31
10 years – 17.9 years	18	32	15	19
Adult (>18 years)	11	12	6	6
**Tumor of Uncertain Malignancy** [3]	-	-	-	3

[1] Status confirmed to best of our knowledge through medical record review, external record update requests, and BWS Registry contact as available.

[2] Years of follow-up from birth until date of last medical record and/or last BWS Registry contact.

[3] Tumors not originally classified as 'typical BWSp tumor' in dataset coding and/or those developed during observation period and were resected due to uncertain malignancy status prior to pathology information. All three patients had positive blood testing and BWSp diagnosis established prior to tumor detection and these tumor types were not malignant. Included two patients personally treated at our institution with adrenal tumors classified as 'adrenal cortical neoplasm' through pathology: a female with IC2 LOM with resection at 2 years, 5 months of age; and a female with pUPD11 with resection at 4 years, 1 month of age. An additional female patient with IC2 LOM enrolled through the BWS Registry had care at another institution for a renal pole mass that was resected at age 2 years, 11 months *(Pathology Report=Hyperlobulated kidney with severe medullary dysplasia and severe interstitial nephritis with extensive global sclerosis of glomeruli. Flanking lobules of renal tissue, no histopathological abnormality).*

[4] June 2021 follow-up was performed within patients at our institution and/or those with BWS Registry contact within the last 3 months.

Table B2. Rates of tumor development associated with BWSp clinical score criteria classification.

**Type of BWSp Tumor** (ICG-BWSp Criteria)	**Cohort Group** (n=215 patients)	**Tumor Type** (n=46 tumors)	**Tumor Types Associated with BWSp Subtypes**
		N=46 tumors	
**'Cardinal Feature' Tumor [1]**	**14 (6.5%)**	**30.4%**	**IC1 GOM (n=10); pUPD11 (n=3); IC2 LOM (n=1)**
Bilateral WT	11	23.9%	IC1 GOM (n=8); pUPD11 (n=2); IC2 LOM (n=1)
Bilateral Nephroblastomatosis	3	6.5%	IC1 GOM (n=2); pUPD11 (n=1)
**'Suggestive Feature' Tumor**	**29 (13.5%)**	**63.0%**	**pUPD11 (n=21); IC1 GOM (n=6); IC2 LOM (n=4)**
Unilateral WT	15	32.6%	pUPD11 (n=8); IC1 GOM (n=6); IC2 LOM (n=1)
Hepatoblastoma (HB)	11 [2]	26.1% [2]	pUPD11 (n=9); IC2 LOM (n=2)
Neuroendocrine Tumors	4	8.7%	pUPD11 (n=3); IC2 LOM (n=1)
Neuroblastoma	2	4.5%	IC2 LOM (n=1); pUPD11 (n=1)
Adrenocortical carcinoma	1	2.2%	pUPD11 [3]
Pheochromocytoma	1	2.2%	pUPD11
Pancreatoblastoma	1	2.2%	pUPD11 [3]

[1] 'Bilateral WT' includes those with WT diagnosed in both kidneys and/or WT in one kidney and bilateral nephroblastomatosis in other kidney; 'Bilateral Nephroblastomatosis' includes those without WT detected/diagnosed in either kidney.

[2] One patient with pUPD11 developed a second primary HB (confirmed through different pathology and somatic testing) – HB (n=12) accounted for within rate. One patient with IC2 LOM had recurrent HB diagnosed multiple times; last follow-up at age 7-8 years (not classified as a new primary in database).

[3] Patients also had history of HB development. Adrenocortical carcinoma diagnosis was pre-HB development and PBL diagnosis was post-HB development and resection.

Table B3. Tumor Phenotype Comparisons within BWSp Study Population.

Population Characteristics	Study Cohort (n=215)	No Tumor (n=172)	Tumor (n=43)	p-value
**Subtype Distributions**				NP
IC1 GOM	30	14	16	
IC2 LOM	112	107	5	
pUPD11	73	51	22	
**Blood+**	82.5%	87.2%	64.3%	0.001**
**Male sex**	44.7%	46.5%	37.2%	0.306
**Diversity Groups**				0.822
'White/Caucasian' only	67.5%	68.1%	65.1%	
'Mixed' race/ethnicity	17.7%	16.9%	20.9%	
'Other' race/ethnicity	14.8%	15.1%	14.0%	
**Conception Type**				0.066 [1]
Natural	80.0%	77.4%	90.2%	
IVF/ICSI	19.0%	22.0% [1]	7.3% [1]	
Other ART	1.0%	0.6%	2.4%	
**Multiple Gestation**	15.1%	16.0%	11.9%	0.633
**ICG-Pregnancy**	34.4%	36.4%	26.3%	0.259
Polyhydramnios	25.9%	27.8%	18.4%	0.302
Placentomegaly	14.4%	15.3%	10.5%	0.607
**Preterm Birth** (<37 weeks)	40.9%	40.5%	42.5%	0.859
**Common Features**				
Macroglossia	71.8%	77.8%	47.6%	<0.001***
BWSp-LO	73.8%	72.0%	81.0%	0.325
Ear creases/pits	62.2%	67.1%	42.1%	0.005**
Facial Nevus Simplex	51.0%	58.5%	20.5%	<0.001***
Large Size (LGA)	63.5%	63.6%	63.2%	1.000
**Organomegaly**	33.7%	31.6%	41.5%	0.267
Nephromegaly	19.6%	16.8%	30.0%	0.074
Hepatomegaly	19.8%	19.6%	20.5%	1.000
Splenomegaly	14.5%	13.6%	17.9%	0.456
**Hypoglycemia**	60.1%	64.2%	43.9%	0.021*
Severe (HI)	20.9%	21.4%	18.6%	0.834
Transient	38.4%	42.0%	24.4%	0.048*
**Abdominal Wall Defects**	70.1%	75.0%	50.0%	0.003**
Omphalocele	24.1%	26.6%	14.0%	0.109
Minor defect	45.1%	47.6%	35.0%	0.162

NP=not performed.
[1] Difference by column proportion testing.

Table B4. Classic BWS Phenotype Population – Tumor Phenotype Comparisons.

Classic BWS Population Characteristics	Classic BWS No Tumor (n=124)	Classic BWS Tumor (n=20)	p-value
**Subtype Distributions**			
IC1 GOM (n=17)	9	6	NP
IC2 LOM (n=94)	90	4	
pUPD11 (n=35)	25	10	
**Blood+**	113/116	20/20	1.000
**Male sex**	60/124	9/20	0.814
**Diversity Groups**			0.113
'White/Caucasian' only	86/120	10/20	
'Mixed' race/ethnicity	21/120	5/20	
'Other' race/ethnicity	13/120	5/20	
**Conception Type**			0.686
Natural	90/119	16/19	
IVF/ICSI	28/119	3/19	
Other ART	1/119	0/19	
**Multiple Gestation**	21/121	4/20	0.756
**ICG-Pregnancy**	52/112	10/18	0.612
Polyhydramnios	41/112	7/18	1.000
Placentomegaly	21/111	4/18	0.751
**Preterm Birth** (<37 weeks)	57/120	16/20	0.008**
**Common Features**			
Macroglossia	116/124	19/20	1.000
BWSp-LO	86/119	15/20	1.000
Ear creases/pits	94/114	12/18	0.123
Facial Nevus Simplex	81/117	7/18	0.017*
Large Size (LGA)	90/122	15/19	0.781
**Organomegaly**	44/115	13/20	0.030*
Nephromegaly	23/109	10/19	0.008**
Hepatomegaly	26/108	6/18	0.394
Splenomegaly	18/107	5/18	0.323
**Hypoglycemia**	82/120	14/20	1.000
Severe (HI)	22/124	7/20	0.128
Transient	60/120	7/20	0.237
**Abdominal Wall Defects**	105/120	15/20	0.166
Omphalocele	43/123	6/20	0.802
Minor defect	62/120	9/20	0.635

NP=not performed.

Table B5. Atypical BWSp / ILO Phenotypes Population – Tumor Phenotype Comparisons.

Atypical BWSp / ILO Population Characteristics	Atypical / ILO No Tumor (n=40)	Atypical / ILO Tumor (n=22)	p-value
**Subtype Distributions**			
IC1 GOM (n=15)	5	10	NP
IC2 LOM (n=12)	11	1	
pUPD11 (n=35)	24	11	
**Blood+**	22/40	6/21	0.062 [1]
**Male sex**	17/40	7/22	0.586
**Diversity Groups**			0.247
'White/Caucasian' only	25/40	17/22	
'Mixed' race/ethnicity	7/40	4/22	
'Other' race/ethnicity	8/40	1/22	
**Conception Type**			0.095
Natural	32/37	20/21	
IVF/ICSI	5/37	0/21	
Other ART	0/37	1/21	
**Multiple Gestation**	5/38	1/21	0.407
**ICG-Pregnancy**	3/38	0/20	0.544
Polyhydramnios	1/38	0/20	1.000
Placentomegaly	2/38	0/20	0.540
**Preterm Birth** (<37 weeks)	9/39	1/20	0.141
**Common Features**			
Macroglossia	11/40	1/22	0.042*
BWSp-LO	29/40	18/21	0.342
Ear creases/pits	8/37	4/20	1.000
Facial Nevus Simplex	9/39	1/21	0.084
Large Size (LGA)	15/39	9/19	0.578
**Organomegaly**	5/38	4/21	0.708
Nephromegaly	2/38	2/21	0.611
Hepatomegaly	3/38	2/21	1.000
Splenomegaly	1/38	2/21	0.286
**Hypoglycemia**	20/39	4/21	0.026*
Severe (HI)	14/40	1/22	0.011*
Transient	6/39	3/21	1.000
**Abdominal Wall Defects**	15/40	5/20	0.395
Omphalocele	1/40	0/22	1.000
Minor defect	14/40	5/20	0.560

NP=not performed.

[1] Difference by column proportion testing.

Table B6. Lateralized Overgrowth Population (BWSp-LO) – Tumor Phenotype Comparisons.

Lateralized Overgrowth (LO) Characteristics	BWSp-LO No Tumor (n=118)	BWSp-LO Tumor (n=34)	p-value
**Subtype Distributions**			<0.001***
IC1 GOM (n=11)	10/118	11/34	
IC2 LOM (n=62)	60/118	2/34	
pUPD11 (n=69)	48/118	21/34	
**Blood+**	96/118	19/34	0.002**
**Male sex**	53/118	14/34	0.845
**Diversity Groups**			0.897
'White/Caucasian' only	76/116	21/34	
'Mixed' race/ethnicity	23/116	7/34	
'Other' race/ethnicity	17/116	6/34	
**Conception Type**			0.152
Natural	87/109	29/32	
IVF/ICSI	21/109	2/32	
Other ART	1/109	1/32	
**Multiple Gestation**	15/112	3/33	0.764
**ICG-Pregnancy**	31/109	7/30	0.649
Polyhydramnios	22/108	5/30	0.797
Placentomegaly	12/108	3/30	1.000
**Preterm Birth** (<37 weeks)	43/112	13/31	0.836
**Common Features**			
Macroglossia	84/118	14/33	0.004**
Ear creases/pits	74/111	15/31	0.092
Facial Nevus Simplex	61/111	7/32	0.001**
Large Size (LGA)	71/115	19/30	1.000
**Organomegaly**	31/107	10/32	0.827
Nephromegaly	16/105	7/32	0.420
Hepatomegaly	21/104	5/31	0.796
Splenomegaly	15/104	4/31	1.000
**Hypoglycemia**	78/113	14/32	0.012*
Severe (HI)	29/117	7/34	0.819
Transient	49/113	7/32	0.039*
**Abdominal Wall Defects**	79/112	12/32	0.003*
Omphalocele	22/116	5/34	0.800
Minor defect	57/112	8/32	0.015*

Table B7. BWSp-Organomegaly Population – Tumor Phenotype Comparisons.

Organomegaly Characteristics	Organomegaly No Tumor (n=49)	Organomegaly Tumor (n=17)	p-value
**Subtype Distributions**			NP
IC1 GOM (n=13)	7	6	
IC2 LOM (n=31)	29	2	
pUPD11 (n=22)	13	9	
**Blood+**	42/47	15/16	1.000
**Male sex**	31/49	4/17	0.010*
**Diversity Groups**			0.043*
'White/Caucasian' only	33/46	7/17	
'Mixed' race/ethnicity	5/46	6/17	
'Other' race/ethnicity	8/46	4/17	
**Conception Type**			0.667
Natural	40/47	15/16	
IVF/ICSI	7/47	1/16	
Other ART	0/47	0/16	
**Multiple Gestation**	11/48	3/17	0.745
**ICG-Pregnancy**	22/44	6/15	0.382
Polyhydramnios	20/44	5/15	0.548
Placentomegaly	9/44	2/15	0.712
**Preterm Birth** (<37 weeks)	24/48	9/17	1.000
**Common Features**			
Macroglossia	43/49	13/17	0.267
BWSp-LO	16/49	10/16	1.000
Ear creases/pits	33/44	7/14	0.102
Facial Nevus Simplex	23/47	3/14	0.122
Large Size (LGA)	34/47	12/15	0.739
**Specific Organs Affected**			
Nephromegaly	25/43	12/16	0.365
Hepatomegaly	29/42	8/15	0.349
Splenomegaly	15/41	4/15	0.543
**Hypoglycemia**	33/49	10/17	0.564
Severe (HI)	10/49	5/17	0.508
Transient	23/49	5/17	0.262
**Abdominal Wall Defects**	41/47	13/16	0.681
Omphalocele	16/49	4/17	0.555
Minor defect	25/47	9/16	1.000

NP=not performed.

Table B8. Wilms Tumor (WT)-Tumor Phenotype Comparisons within BWSp Population.

Population Characteristics	No Tumor (n=172)	BWSp-WT (n=29)	p-value
**Subtype Distribution**			
IC1 GOM (n=30)	14	16	
IC2 LOM (n=109)	107	2	NP
pUPD11 (n=62)	51	11	
**Blood+**	143/164	15/28	<0.001***
**Male sex**	80/172	10/29	0.313
**Diversity Groups**			0.879
'White/Caucasian' only	113/166	19/29	
'Mixed' race/ethnicity	28/166	6/29	
'Other' race/ethnicity	25/166	4/29	
**Conception Type**			0.081 [1]
Natural	123/159	25/28	
IVF/ICSI	35/159	2/28	
Other ART	1/159	1/28	
**Multiple Gestation**	26/163	2/28	0.383
**ICG-Pregnancy**	55/151	5/27	0.080
Polyhydramnios	42/151	3/27	0.091
Placentomegaly	23/150	2/27	0.377
**Preterm Birth** (<37 weeks)	66/163	8/27	0.394
**Common Features**			
Macroglossia	133/171	10/29	<0.001***
BWSp-LO	118/164	21/28	0.823
Ear creases/pits	104/155	7/26	<0.001***
Facial Nevus Simplex	93/159	4/27	<0.001***
Large Size (LGA)	105/165	14/26	0.386
**Organomegaly**	49/155	10/28	0.666
Nephromegaly	25/149	7/27	0.280
Hepatomegaly	29/148	6/27	0.795
Splenomegaly	20/147	6/27	0.248
**Hypoglycemia**	104/162	10/28	0.006**
Severe (HI)	36/168	1/29	0.020*
Transient	68/162	9/28	0.406
**Abdominal Wall Defects**	123/164	9/27	<0.001***
Omphalocele	45/169	0/29	<0.001***
Minor defect	78/164	9/27	0.212

NP=not performed.
[1] Difference by column proportion testing.

Table B9. Hepatoblastoma (HB)-Tumor Phenotype Comparisons within BWSp Population.

Population Characteristics	No Tumor (n=172)	BWSp-HB (n=11)	p-value
**Subtype Distribution**			
IC1 GOM (n=14)	14	-	NP
IC2 LOM (n=109)	107	2	
pUPD11 (n=60)	51	9	
**Blood+**	143/164	11/11	0.365
**Male sex**	80/172	5/11	1.000
**Diversity Groups**			0.611
'White/Caucasian' only	113/166	6/11	
'Mixed' race/ethnicity	28/166	3/11	
'Other' race/ethnicity	25/166	2/11	
**Conception Type**			0.640
Natural	123/159	9/10	
IVF/ICSI	35/159	1/10	
Other ART	1/159	0/10	
**Multiple Gestation**	26/163	2/11	0.691
**ICG-Pregnancy**	55/151	4/8	0.470
Polyhydramnios	42/151	3/8	0.688
Placentomegaly	23/150	2/8	0.613
**Preterm Birth** (<37 weeks)	66/163	8/10	0.020*
**Common Features**			
Macroglossia	133/171	9/10	0.692
BWSp-LO	118/164	11/11	0.069[1]
Ear creases/pits	104/155	9/9	0.058[1]
Facial Nevus Simplex	93/159	4/9	0.496
Large Size (LGA)	105/165	8/9	0.163
**Organomegaly**	49/155	6/10	0.085
Nephromegaly	25/149	4/10	0.085
Hepatomegaly	29/148	2/9	1.000
Splenomegaly	20/147	1/9	1.000
**Hypoglycemia**	104/162	7/10	1.000
Severe (HI)	36/168	6/11	0.022*
Transient	68/162	1/10	0.052[1]
**Abdominal Wall Defects**	123/164	9/10	0.454
Omphalocele	45/169	5/11	0.182
Minor defect	78/164	4/10	0.751

NP=not performed.

[1] Difference by column proportion testing.

Table B10. IC1 GOM Population – Tumor Phenotype Comparisons.

IC1 GOM Characteristics	IC1 GOM – No Tumor (n=14)	IC1 GOM Tumor (n=16)	p-value
**Blood+**	13/14 [1]	9/15 [1]	0.080 [1]
**Male sex**	7/14	5/16	0.457
**Diversity Groups**			0.297
'White/Caucasian' only	11/13	10/16	
'Mixed' race/ethnicity	2/13	4/16	
'Other' race/ethnicity	0/13	2/16	
**Conception Type**			0.669
Natural	11/12	14/16	
IVF/ICSI	0/12	1/16	
Other ART	1/12	1/16	
**Multiple Gestation**	2/13	1/15	0.585
**ICG-Pregnancy**	7/13	2/14	0.046*
Polyhydramnios	6/13	0/14	0.006**
Placentomegaly	1/13	2/14	1.000
**Preterm Birth** (<37 weeks)	6/13	6/15	1.000
**Common Features**			
Macroglossia	10/14	7/16	0.159
BWSp-LO	10/13	11/15	1.000
Ear creases/pits	4/12	4/13	1.000
Facial Nevus Simplex	2/11	3/14	1.000
Large Size (LGA)	8/13	9/13	1.000
**Organomegaly**	7/12	6/15	0.449
Nephromegaly	5/12	3/14	0.401
Hepatomegaly	5/12	4/14	0.683
Splenomegaly	4/12	1/14	0.148
**Hypoglycemia**	8/13	6/16	0.272
Severe (HI)	3/14	0/16	0.090
Transient	5/13	6/16	1.000
**Abdominal Wall Defects**	11/14 [1]	5/14 [1]	0.054 [1]
Omphalocele	0/14	0/16	n/a
Minor defect	11/14 [1]	5/14 [1]	0.054 [1]

n/a=not applicable.

[1] Difference by column proportion testing.

Table B11. Tumor Phenotype Comparisons within IC2 LOM Population.

IC2 LOM Characteristics	IC2 LOM – No Tumor (n=107)	IC2 LOM Tumor (n=5)	p-value
**Blood+**	99/101	5/5	1.000
**Male sex**	49/107	2/5	1.000
**Diversity Groups**			0.343
'White/Caucasian' only	71/102	5/5	
'Mixed' race/ethnicity	18/102	0/5	
'Other' race/ethnicity	13/102	0/5	
**Conception Type**			0.652
Natural	70/102	3/5	
IVF/ICSI	32/102	2/5	
Other ART	0/102	0/5	
**Multiple Gestation**	19/103 [1]	3/5 [1]	0.056 [1]
**ICG-Pregnancy**	39/91	4/5	0.170
Polyhydramnios	30/91	3/5	0.335
Placentomegaly	18/90	1/5	1.000
**Preterm Birth** (<37 weeks)	49/102	4/5	0.205
**Common Features**			
Macroglossia	103/107	4/5	0.208
BWSp-LO	60/101	2/5	0.647
Ear creases/pits	78/96 [1]	2/5 [1]	0.059 [1]
Facial Nevus Simplex	74/100	2/5	0.128
Large Size (LGA)	66/103	2/5	0.357
**Organomegaly**	29/97	2/5	0.638
Nephromegaly	14/92	2/5	0.189
Hepatomegaly	13/91	0/5	1.000
Splenomegaly	10/90	2/5	0.118
**Hypoglycemia**	56/100	3/5	1.000
Severe (HI)	12/105	1/5	0.473
Transient	44/100	2/5	1.000
**Abdominal Wall Defects**	84/101	5/5	1.000
Omphalocele	43/107	3/5	0.400
Minor defect	41/101	2/5	1.000

[1] Difference by column proportion testing.

Table B12. Tumor Phenotype Comparisons within pUPD11 Population.

pUPD11 Population Characteristics	pUPD11 No Tumor (n=51)	pUPD11 Tumor (n=22)	p-value
**Blood+**	31/49	13/22	0.795
**Male sex**	24/51	9/22	0.798
**Diversity Groups**			0.728
'White/Caucasian' only	31/51	13/22	
'Mixed' race/ethnicity	8/51	5/22	
'Other' race/ethnicity	12/51	4/22	
**Conception Type**			0.547
Natural	42/45	20/20	
IVF/ICSI	3/45	0/20	
Other ART	0/45	0/20	
**Multiple Gestation**	5/47	1/22	0.656
**ICG-Pregnancy**	9/47	4/19	1.000
Polyhydramnios	6/47	4/19	0.456
Placentomegaly	4/47	1/19	1.000
**Preterm Birth** (<37 weeks)	11/48	7/20	0.370
**Common Features**			
Macroglossia	20/50	9/21	1.000
BWSp-LO	48/50	21/22	1.000
Ear creases/pits	22/47	10/20	1.000
Facial Nevus Simplex	17/48	3/20	0.144
Large Size (LGA)	31/49	13/20	1.000
**Organomegaly**	13/46	9/21	0.271
Nephromegaly	6/45	7/21	0.094
Hepatomegaly	11/45	4/20	0.761
Splenomegaly	6/45	4/20	0.482
**Hypoglycemia**	40/49	9/20	0.004**
Severe (HI)	21/49	7/22	0.439
Transient	19/49	2/20	0.022*
**Abdominal Wall Defects**	28/49	10/21	0.602
Omphalocele	2/48	3/22	0.316
Minor defect	26/49	7/21	0.192

Table B13. Wilms Tumor (WT)-Tumor Phenotype Comparisons within pUPD11 Population.

pUPD11 Population Characteristics	pUPD11 No Tumor (n=51)	pUPD11-WT (n=11)	p-value
**Blood+**	31/49	4/11	0.174
**Male sex**	24/51	5/11	1.000
**Diversity Groups**			0.922
'White/Caucasian' only	31/51	7/11	
'Mixed' race/ethnicity	8/51	2/11	
'Other' race/ethnicity	12/51	2/11	
**Conception Type**			1.000
Natural	42/45	10/10	
IVF/ICSI	3/45	0/10	
Other ART	0/45	0/10	
**Multiple Gestation**	5/47	0/11	0.572
**ICG-Pregnancy**	9/47	2/11	1.000
Polyhydramnios	6/47	2/11	0.639
Placentomegaly	4/47	0/11	1.000
**Preterm Birth** (<37 weeks)	11/48	1/10	0.670
**Common Features**			
Macroglossia	20/50	2/11	0.299
BWSp-LO	48/50	10/11	0.455
Ear creases/pits	22/47	3/11	0.320
Facial Nevus Simplex	17/48	0/11	0.024*
Large Size (LGA)	31/49	5/11	0.321
**Organomegaly**	13/46	3/11	1.000
Nephromegaly	6/45	3/11	0.358
Hepatomegaly	11/45	2/11	1.000
Splenomegaly	6/45	4/11	0.093
**Hypoglycemia**	40/49	3/10	0.002**
Severe (HI)	21/49	1/11	0.043*
Transient	19/49	2/10	0.470
**Abdominal Wall Defects**	28/49	2/11	0.042*
Omphalocele	2/48	0/11	1.000
Minor defect	26/49	2/11	0.048*

Table B14. Hepatoblastoma (HB)-Tumor Phenotype Comparisons within pUPD11 Population.

pUPD11 Population Characteristics	pUPD11 No Tumor (n=51)	pUPD11-HB (n=9)	p-value
**Blood+**	31/49	9/9	0.045*
**Male sex**	24/51	3/9	0.495
**Diversity Groups**			0.437
'White/Caucasian' only	31/51	4/9	
'Mixed' race/ethnicity	8/51	3/9	
'Other' race/ethnicity	12/51	2/9	
**Conception Type**			1.000
Natural	42/45	8/8	
IVF/ICSI	3/45	0/8	
Other ART	0/45	0/8	
**Multiple Gestation**	5/47	1/9	1.000
**ICG-Pregnancy**	9/47	2/6	0.592
Polyhydramnios	6/47	2/6	0.219
Placentomegaly	4/47	1/6	0.465
**Preterm Birth** (<37 weeks)	11/48	6/8	0.007**
**Common Features**			
Macroglossia	20/50	7/8	0.020*
BWSp-LO	48/50	9/9	1.000
Ear creases/pits	22/47	7/7	0.012*
Facial Nevus Simplex	17/48	3/7	0.696
Large Size (LGA)	31/49	6/7	0.403
**Organomegaly**	13/46	6/8	0.017*
Nephromegaly	6/45	4/8	0.033*
Hepatomegaly	11/45	2/7	1.000
Splenomegaly	6/45	0/7	0.580
**Hypoglycemia**	40/49	5/8	0.345
Severe (HI)	21/49	5/9	0.717
Transient	19/49	0/8	0.042*
**Abdominal Wall Defects**	28/49	7/8	0.134
Omphalocele	2/48	3/9	0.024*
Minor defect	26/49	4/8	1.000

Table B15. Patients selected for series: Results from 2019 BWS Registry cohort and 2020 BWSp Research Network database queries.

Patient ID	Database Identified	Search Criteria Matched [1]					Criteria Match Count	Profile Eligibility		
		A	B	C	D	E		BWSp Phenotype	BWSp Epigenotype	Cancer Type Previously Reported [2]
#01	2019 cohort	+	+	-	-	+	3/5	+	+	+
#02	2019 cohort	+	+	+	+	-	4/5	+	+	+
#03	2019 cohort	+	-	-	+	-	2/5	+	+	Unreported (New)
#04	BWSp Network	+	+	-	+	-	3/5	+	+	+
#05	2019 cohort	+	-	-	-	-	1/5	+	+	+
#06	BWSp Network	+	+	-	-	-	2/5	L	+	+
#07	2019 cohort	-	+	-	-	+	2/5	+	+	+
#08	2019 cohort	-	+	-	-	+	2/5	+	+	+
#09	2019 cohort	-	+	-	-	+	2/5	+	+	+
#10	BWSp Network	-	+	-	-	-	1/5	+	+	+
#11	2019 cohort	-	+		-	-	1/4	L	+	+
#12	2019 cohort	-	+	-	-	-	1/5	+	+	Unreported (New)
#13	2019 cohort	-	-	+	-	-	1/5	+	+	+
#14	BWSp Network	-	-	+		-	1/4	L	+	+
#15	BWSp Network	-	+	-		-	1/4	L	+	+
#16	BWSp Network	+	+	-	+	-	3/5	+	+	Unreported (New)
#17	2019 cohort	-	-	+	+	-	2/5	+	+	+
#18	BWSp Network	+	+	-	-	-	2/5	+	+	Unreported (New)
#19	2019 cohort	-	+	-	+	+	3/5	+	+	+
#20	BWSp Network	-	-	+	+	-	2/5	+	+	+
#21	2019 cohort	-	-	-	-	++	1/5	+	+	Unreported (New)
#22	2019 cohort	+	-	+	+	-	3/5	+	R	+
#23	2019 cohort	-	+	-	-	+	2/5	+	L	+
#24	2019 cohort	-	+	-	-	-	1/5	+	L	+
#25	2019 cohort	-	+	-	-		1/4	+	R	+
#26	2019 cohort	-	-	-	-	+	1/5	+	R	+

[1] Search Criteria: A=Tumor-IC2 LOM-tumor; B=HB tumor type; C=Tumor-ART conception; D=Tumor-Multiple gestation; E=Tumor-Hyperinsulinism (HI).

[2] Patients previously included in cohorts evaluating HB or WT in BWSp marked with (+); Tumor types or patients with HB or WT not included in previous cohorts are highlighted (New).

Table Abbreviations and Symbols: (+)=Yes; (-)=No; L=Limited (incomplete); R=Reported (outside labs).

Table B16. Common Characteristics and Phenotype Patterns Observed in Selected Patients with BWSp and Tumor Development.

ID	ART and/or Twinning Hx [1]	BWSp Diagnosis Pre-Tumor	Organ and/or GU Anomalies	Macroglossia Severity [2]	Craniofacial Features	Abdominal Wall Defect (AWD)	Hypoglycemia Severity	LO [3]
#01	- / -	+	+	++	+	Omphalocele	HI	+
#02	+ / +	+	+	+++	+	Omphalocele	Transient	+
#03	- / +	+	+	++	+	Omphalocele	No	No
#04	- / +	+	-	+	+	Umbilical Hernia	No	No
#05	+Fam Hx / -	Post-WT	Not known prior to WT	No	No	Umbilical Hernia	No	No
#07	- / -	+	+	+++	+	Omphalocele	HI	+++
#08	+Fam Hx / -	+	+	+++	+	Umbilical Hernia	HI	+++
#09	- / -	+	+	+++	+	Umbilical cord cyst (in-utero)	HI	+++
#10	- / +	+	No	No	+	Umbilical Hernia	Transient	++
#12	- / -	C [4]	Not known prior to HB	+	+	Umbilical Hernia	No	+
#13	+ / -	+	No	+	+	No	No	++
#16	**- / +**	Post-HB	Not known prior to HB	No	+	No	No	+
#17	**+ / +**	Post-WT	+	?*	+	Umbilical Hernia	No	+
#18	**- / -**	+	-	+	+	No	No	+
#19	**- / +**	+	+	+++	+	Omphalocele	HI	+++
#20	**+ / +**	+	+	No	+	No	No	++
#21	**- / -**	C [1]	No	No	+	No	HI (Panc) [5]	+
#22	**+ / +**	Post-WT	+	+	+	Diastasis Recti	Transient	No
#23	**- / -**	+	+	++	+	Umbilical Hernia	HI	+++
#24	**- / +**	+	No	No	+	Umbilical Hernia	Transient	+++
#25	**- / -**	+	+	++	+	Omphalocele	unknown	+++
#26	**- / -**	+	+	++	+	Umbilical Hernia	HI	No

[1] History of ART conception or twinning (+)yes, (-)no; Patients #05 and #08 (females) were naturally conceived, but had an older brother conceived using ART (no family history of BWSp for either patients).
[2] Lateralized Overgrowth (LO) Severity Scale: (+)=Mild asymmetry; (++)=Obvious asymmetry and/or LLD (leg length discrepancy); (+++)=Severe asymmetry/LO.
[3] Macroglossia Severity Scale: (+)=Mild macroglossia *(no surgery needed or projected at last follow-up)*; (++)=Macroglossia requiring tongue reduction (TR) >1 year of age; (+++)=Severe macroglossia (TR < 1 year of age required for management).
[4] C = concordant with suspected BWSp diagnosis (HB detected on first screen during diagnostic work-up).
[5] HI (Panc)=Severe Hyperinsulinism requiring pancreatectomy for management.

Table B17. Summary of Common Characteristics Observed in Patients with Twinning or Higher Order Gestation.

Patient ID	Conception	Twinning	Major Pregnancy Complications	Birth Outcome	Gest. Age (weeks)	BWSp Diagnosis	Subtype	Tumor
**Female Twins or Triplets**								
#**03** (Female)	Natural	MZ Triplets (monochor-triamniotic)	TRAP sequence (proband=donor to acardic fetus)	Discordant MZ Twins +PMD	27 $^{3/7}$ wks	Prenatal	IC2 LOM (blood)	NBL
#**04** (Female)	Natural	Triplets MZ twins/Singleton (Dichor-Triamniotic)	TTTS of MZ twins (proband=donor) IUFD of twin (23 wks)	DZ Twins	30 $^{6/7}$ wks	Birth	IC2 LOM (blood)	HB
#**16** (Female)	Natural	MZ Twins (monochorionic-diamniotic)	TTTS @ 23 wks (proband=recipient) IUFD of twin (25 wks)	Singleton GA=26 4/7 wks	26 $^{4/7}$ wks	Post-HB	IC2 LOM (tissue)	HB
#**22** (Female)	IVF	MZ Twins (diamniotic)	TTTS @19-20 wks (proband=recipient) Pre-Eclampsia	Discordant MZ Twins (+CAKUT in proband)	35 $^{3/7}$ wks	Post-WT	IC2 LOM (blood)	WT
**Male-Male Twins**								
#**02** (Male)	Clomp stim +IVF-ICSI (donor egg)	Twins (Discordant)	Bleeding, Pre-Eclampsia, Placenta Previa +Polyhydramnios	Discordant Twins GA= 28 6/7	28 $^{6/7}$ wks	Prenatal confirmation	IC2 LOM (blood)	HB
#**17** (Male)	Clomp stim	Twins (di-di)	Hydronephrosis (proband @24 wks) Pre-Eclampsia No Polyhydramnios	Discordant Twins (+CAKUT in proband)	35 $^{5/7}$ wks	Post-WT	IC1 GOM (tissue)	WT
#**19** (Male)	Natural	Twins (Discordant)	Limited prenatal history available (+Omphalocele)	Discordant Twins	33 wks	Birth	pUPD11 (blood)	HB
**Female-Male Twins**								
#**20** (Female)	IVF	Twins (Discordant)	No issues reported	Discordant Twins	34 wks	Infancy (LO)	IC1 GOM (tissue)	WT
**Unknown Twin Sex**								
#**10** (Male)	Natural	Vanishing embryo	No major issues (LGA)	Singleton +Placentomegaly	39 $^{3/7}$ wks	Infancy (LO+)	pUPD11 (blood)	HB
#**24** (Female)	Natural	Empty sac[1]	No major issues (LGA)	Singleton	38 wks	Neonatal (LO+)	pUPD11 (blood)	HB

[1] Patient's direct older siblings are twins (male-female) without BWSp.
Uncommon Table Abbreviations: CAKUT=congenital anomality of the kidney and/or urinary tract; IUFD=intrauterine fetal demise; MZ=monozygotic; TTTS=Twin-Twin Transfusion Syndrome; TRAP=Twin Reversed Arterial Perfusion (TRAP) Sequence

Table S1. Characteristics Associated with Multiple Tumor Development in Patients with BWSp due to pUPD11.

	Patient #07	Patient #08	Patient #19
**Demographic Characteristics**			
Patient Sex	Female	Female	Male
Diversity Group	Mixed race/ethnicity	White (non-Hispanic)	Mixed race/ethnicity *(suspected)*
**Prenatal Phenotype**	Severe BWSp	Severe Anomalies	Severe BWSp
Prenatal Nephromegaly	Yes	Yes	Unknown [1]
**Conception Profile**	Natural conception	Natural conception<br>+ART in older brother	Natural (assumed)
**Twinning**	No report of vanishing twin	No report of vanishing twin	Male Twins (discordant)
**Gestational Age**	32 $^{1/7}$ weeks	33 $^{1/7}$ weeks	33 weeks
**11p15 Cell Burden** (Blood)	65-70% pUPD11	~65% pUPD11	50-55% pUPD11
**BWSp Clinical Score**	11 (Severe BWSp)	11 (Severe BWSp)	12 (Severe BWSp)
**Abdominal Wall Defect**	Omphalocele	Umbilical Hernia	Omphalocele
**Hyperinsulinism (HI)**	Yes (resolved)	Yes (resolved)	Severe (persistent)
**Macroglossia Profile**	Severe - Thick<br>(TR age <1 year)	Severe - Thick<br>(TR age <6 months)	Severe - Thick<br>(three TRs)
**Lateralized Overgrowth**	Severe (Full Body)<br>Right > Left	Severe (Full Body)<br>Left > Right	Yes (Full Body)<br>Left > Right
Largest Girth Difference [2]	~25% (Thighs)	~15% (Forearms)	~5-10% (Forearm, Palms)
**Neonatal Kidney Findings**	Echogenic with diffuse bilateral enlargement (possible microcysts)	Asymmetric kidney size with bilateral fetal lobulation (no masses)	Echogenic and enlarged (cystic)
**Hepatoblastoma (HB) Tumor Development**			
Age at First HB Detection	**6 months (HB)**	**2 months (HB)**	**4 months (HB)**
Diagnosis Comments	*3 distinct lesions*	*Large solid mass*	*3 distinct lesions*
Second HB Detection Characteristics	**No additional HB developed through last follow-up**	**14 months (HB)**<br>Second Primary<br>*(~1 year post initial HB treatment)*	**31 months (HB)**<br>Recurrence<br>*(~2 years post initial HB resection)*
**Other Tumor Development**			
Age and Type of Tumor	**8.5 months**<br>**Pancreatoblastoma**	**No other tumors developed through last follow-up**	**3 months**<br>**Adrenocortical carcinoma**
Diagnosis Comments	*2 distinct lesions; No specific treatment required (calcified)*		*Adrenal pseudocyst and adrenal cortex cytomegaly also diagnosed through pathology*
**Age at Last Follow-Up**	**3 years, 1 month**	**4 years, 7 months**	**6 years, 6 months**
Patient Status	Alive and Well (NED)	Alive and Well (NED)	Alive and Well (NED)

[1] Patient was adopted – limited prenatal history is known; Twin not affected by BWSp *(long-term outcome unknown)*.
[2] Girth asymmetry assessed by absolute value between difference of circumference at reference point in each limb compared to the smaller limb size *(assumed limb without 'overgrowth')*.
Table Abbreviations: ART=Assisted Reproductive Technique; BWSp=Beckwith-Wiedemann Spectrum; HB=Hepatoblastoma; NED=No Evidence of Disease; pUPD11=paternal uniparental isodisomy of chromosome 11; TR=Tongue Reduction.

Table S2. Epigenotype and Somatic HB Profiles Established in Samples Collected from Patients with pUPD11 and Multiple Tumor Development.

Samples Analyzed [1]	SNP Testing	Methylation (IC2 region)	Methylation (IC1 region)	11p15 Mosaic Burden	Profile Established
**Patient #07 (Female)**					
Blood	65-70% LOH	13.06% (LOM)	80.39% (GOM)	67.30%	**pUPD11** (11p15.5p15.2)
Skin Biopsy	40% LOH	-	-	-	
**Patient #08 (Female)**					
Blood	~65% LOH	16.87% (LOM)	80.27% (GOM)	63.40%	**pUPD11** (11p15.5)
Skin Biopsy	~30% LOH	-	-	-	
Normal Liver (HB1)	-	13.43% (LOM)	81.92% (GOM)	68.49%	
First Primary (HB1)	-	1.60% (LOM)	90.54% (GOM)	88.94%	
**Patient #19 (Male)**					
Blood	50-55% LOH	+LOM (OSH)	+GOM (OSH)	N/A	**pUPD11** (11p15.5p14.1)
Skin Biopsy	30-35% LOH	35.45% (LOM)	64.83% (GOM)	29.38%	
Tongue	40-45% LOH	30.87% (LOM)	69.84% (GOM)	38.97%	

Tumor Testing [2]	Copy Number Variation (CNV)	Somatic Sequence Variants [3]
**Patient #07** (HB)	None detected within limits of assay	***CTNNB1***, c.100G>A (p.Gly34Arg)<br>***ARID1A***, c.6698_6704del (p.Arg2233Profs*32)
**Patient #08** (HB1)	None detected within limits of assay	***CTNNB1***, c.98C>T (p.Ser33Phe)
**Patient #08** (HB2) [4]	Loss of partial chromosome 1p<br>Possible low-level mosaic gain of partial chr 1q	***CTNNB1***, c.101G>T (p.Gly34Val)

[1] Blood samples collected pre-tumor detection in Patient #07 and #08, and post-tumor detection in Patient #19. Skin samples collected during surgical procedures for patient care and specific source of skin biopsy included: omphalocele repair site (#07); left abdomen (#08); and left thigh (#19).
[2] Testing on HB samples included next generation sequencing (NGS) analysis of genes in the Children's Hospital of Philadelphia (CHOP) Comprehensive Solid Tumor NGS Panel performed on DNA and RNA extracted from tumor sample; The genes included in the panel and reference sequences used for these genes are listed at: https://apps.chop.edu/service/laboratories/olsd.cfm/division-genomic-diagnostics. The mutation nomenclature is based on the convention recommended by the Human Genome Variation Society (http://www.hgvs.org/mutnomen/).
[3] Variants classified as 'Potential Clinical Significance' listed; Reference/Isoforms: *CTNNB1* (NM_001904.3); *ARID1A* (NM_006015.5)
[4] Second HB detected approximately one year after first HB in patient. Classified as a second primary HB due to different somatic variants: no CNVs were detected in first sample and two different *CTNNB1* variants were detected; differing pathology characteristics compared to first tumor were also observed.

Table Abbreviations: ART=Assisted Reproductive Technique; BWSp=Beckwith-Wiedemann Spectrum; GOM=Gain of Methylation; HB=Hepatoblastoma; IC1= Imprinting Control region 1 (*H19/IGF2*:IG-DMR); IC2= Imprinting Control region 2 (*KCNQ1OT1*:TSS-DMR); LOH=Loss of Heterozygosity; LOM=Loss of Methylation; N/A=Not Applicable; OSH=Outside hospital/laboratory; pUPD11=paternal uniparental isodisomy of chromosome 11; SNP= Single nucleotide polymorphism array testing.

Table S3. Comparison of Clinical Characteristics and Hepatoblastoma (HB) Development History in Two Male Patients with IC2 LOM.

	**Patient #01**	**Patient #02**
**Patient Characteristics**		
**Patient Sex**	Male	Male
**Diversity Group**	White (non-Hispanic)	White (non-Hispanic)
**Blood Epigenotype Profile** (cell burden)	IC2 LOM (99.90%)	IC2 LOM (98.02%)
**BWSp Clinical Score** (Group)	14 (Severe)	10 (Severe)
**Peri-Conception and Prenatal Profiles**		
**Conception Type**	Natural conception	IVF-ICSI (Clomp stim + egg donor)
**Twinning History**	No report of vanishing twin, etc	Male Twins (discordant)
**Prenatal Phenotype**	**Severe BWSp**<br>(+Omphalocele, +Enlarged Kidneys/Liver)	**Severe BWSp**<br>(+Omphalocele, +Macroglossia)
**Pregnancy Complications**	Premature Rupture of Membranes (PROM)<br>No Polyhydramnios	Bleeding, Pre-Eclampsia<br>+ Polyhydramnios
**Placenta Characteristics Known**	Severe Placentomegaly (trimmed weight=882 grams)<br>Placental Mesenchymal Dysplasia (PMD)	Placenta Previa
**Gestational Age at Birth**	34 $^{6/7}$ weeks	28 $^{6/7}$ weeks
**Common BWSp Features**		
Macroglossia Severity	TR age: ~1 year *(no tracheostomy)*	TR age: 6 months *(+ tracheostomy)*
Hypoglycemia Severity	Hyperinsulinism (resolved)	Transient
Lateralized Overgrowth	Yes (mild)	Yes (+LLD)
**Other Phenotypic Features**		
Genitourinary (GU) Abnormalities	Undescended Testes and Inguinal Hernia	Undescended Testes and Inguinal Hernia
Gastrointestinal (GI) Issues	Gastroesophageal reflux disease (GERD)	Gastrotomy Tube Dependence
Cardiopulmonary Issues	Critical pulmonary stenosis requiring valvuloplasty	Pulmonary hypertension requiring tracheostomy
Other Birth Issues	Sacral dimple	Bronchopulmonary Dysplasia
**Hepatoblastoma Diagnosis**		
Age at First Detection	**6 weeks** (screening: AFP + Abd U/S)	**11-12 months** (screening: AFP + Abd U/S)
Detection Comments	Solitary well-circumscribed hypovascular mass [1]<br>(Pathology=Stage 1 (COG))	Large right lobe mass<br>(formal lobectomy)
Alpha-Fetoprotein (AFP) Trend	Rapid increase [2] after imaging detection<br>and close follow-up (decreased from birth value)	Rising values
**Additional HB Development:**<br>Characteristics and Age at Detection	**No HB or other tumors developed through last follow-up**	**25 months**<br>Metastatic lesion<br>(local left lobe resection)<br>**6 years, 2 months**<br>Single lesion in lateral left lobe<br>(hepatic resection)
**Age at Treatment Completion**	**5-6 months**	**6 years, 4 months**
**Age at Last Follow-Up**	**6 years, 4 months**	**10 years, 8 months**
Patient Status	Alive and Well (NED)	Alive and Well (NED)

[1] Impression of mass through MRI (magnetic resonance imaging) was thought to represent atypical hemangioma without classic vascular enhancement; however, HB could not be completely excluded. Clinical correlation with rising AFP values led to biopsy which confirmed HB and upfront resection was performed. Pathology was consistent with Children's Oncology Group (COG) Stage 1 HB.

[2] Alpha-fetoprotein (AFP) values measured in patient throughout work-up, resection, and last follow-up include: 323,000 ng/mL (birth-normal imaging); 3,530 ng/mL (imaging detection at 6 weeks); 3,930 ng/mL (4 days later); 6,650 ng/m (1 week later); 9,910 ng/mL (pre-biopsy, 3 weeks post-imaging); 2,690 ng/mL (post-resection); 133 ng/mL (treatment completion); 3.7 ng/mL (last AFP performed at 5 years, 1 month of age).

Table Abbreviations: Abd U/S=Ultrasound (full abdominal); BWSp=Beckwith-Wiedemann Spectrum; Clomp stim=clomiphene stimulation of ovaries; HB=Hepatoblastoma; IC2 LOM=Loss of methylation at Imprinting Control region 2 (*KCNQ1OT1*:TSS-DMR); NED=No Evidence of Disease; LLD=Leg Length Discrepancy; TR=Tongue Reduction.

Table S4. Additional Evidence for Tissue Mosaicism Associated with ART, IC1 GOM, and WT Development.

**Patient #17 (Male)**	**Characteristics and Phenotype Profiles** [1]
**Prenatal Profile:**	ART (Clomiphene stimulation); Twin Males (di-di); Pre-Eclampsia
**Birth Phenotype:**	GA=35 5/7 weeks; Hydronephrosis (screening initiated); Heart murmur (transient)
**Craniofacial Profile:**	Mild tongue protrusion (normal thyroid) [2]; Ear creases/pits
**GU Phenotype:**	Prenatal asymmetric hydronephrosis (diagnosed 24 weeks gestation, confirmed at birth)
**BWSp Phenotype:**	Post-WT appreciated to have LO and history of umbilical hernia
**Other Notable:**	G6PD deficiency in both twins (glucose-6-phosphate-dehydrogenase deficiency)
**Tumor Presentation:**	Incidental detection during routine imaging for GU anomaly

**Samples Analyzed**[3]	**11p15 Copy Number**	**IC2 Methylation**	**IC1 Methylation**	**Summary**
**Kidney** (normal)	KCNQ1 duplication<br>Normal IC1 (2 copies)	50.18% (N)	82.37% (GOM)	+11p15 (IC1 GOM)<br>Mosaic Burden: 64.74%
**Kidney** (WT)	KCNQ1 duplication<br>Normal IC1 (2 copies)	49.11% (N)	88.00% (GOM)	+11p15 (IC1 GOM)<br>Mosaic Burden: 76.00%
**Blood** (post-WT)	N/A	48.52% (N)	52.33% (N)	No 11p15 detectable<br>Mosaic Burden: <3%

**Patient #20 (Female)**	**Characteristics and Phenotype Profiles** [4]
**Prenatal Profile:**	IVF conception; Twin (female-male); No issues (GA=34 weeks)
**BWSp Phenotype:**	LO, ear crease, nephromegaly (4 points; Atypical/ILO); Male twin=no BWSp
**Craniofacial Profile:**	Mild frontal bossing
**GU Phenotype:**	Nephromegaly with asymmetry diagnosed on initial scan and on follow-ups
**Tumor Presentation:**	Symptomatic with detection on imaging<br>Post-BWSp screening program (17 months after normal imaging at 7 years, 10 months)

**Samples Analyzed** [5]	**SNP Array Result**	**IC2 Methylation**	**IC1 Methylation**	**Summary**
**Blood** (7mo)	Normal Female	Normal	Normal	No 11p15 detected (OSH)<br>Mosaic Burden: N/A
**Skin** (left abdomen)	Normal Female	49.61% (N)	60.26% (GOM)	+11p15 (IC1 GOM)<br>Mosaic Burden: 20.52%
**Kidney** (normal)	Normal Female	50.96% (N)	83.62% (GOM)	+11p15 (IC1 GOM)<br>Mosaic Burden: 67.24%
**Kidney** (WT)	Normal Female	48.47% (N)	66.88% (GOM)	+11p15 (IC1 GOM)<br>Mosaic Burden: 33.76%

[1] Additional characteristics and phenotype data for Patient 9 described by MacFarland et al. (2018), PMID: 29932284.
[2] Classified with macroglossia on initial report – retrospective review demonstrated no official 'macroglossia' diagnosis was made and patient had history of mild tongue protrusion during first year of life (normal tongue at physical examinations post-tumor).
[3] Kidney samples collected during tumor resection at age 33m; Blood sample collected at age 6.5 years (new data collected since MacFarland et al.)
[4] Additional characteristics and phenotype data for patient described by Fischer et al. (2021), PMID: 33581236.
[5] Skin and kidney samples collected at time of tumor resection at approximately 9.5 years of age *(3 months post-WT diagnosis and initial treatment)*; Skin sample collected from surgical incision margin on LO side of patient (full body L>R asymmetry).

Table S5. Evidence for Tissue Mosaicism Associated with Neuroblastoma (NBL) Development in Two Patients with BWSp and Epigenotypes other than *CDKN1C* mutations.

**Patient #21 (Male)**	**Characteristics and Phenotype Profiles**
**Prenatal Profile:**	Natural conception; Singleton; Pre-Eclampsia and LGA
**BWSp Phenotype:**	HI, LO, LGA (5 points; Atypical/ILO); *Possible neonatal FNS (resolved by 7 weeks of age)*
**Craniofacial Profile:**	Slightly high palate (no cleft); Ears upturned with squared off helices (no creases); Deep-set eyes
**GU Phenotype:**	No kidney or liver abnormalities on imaging; No hepatosplenomegaly by palpation
**NBL Tumor Presentation:**	Incidental Detection (CT scan for other medical issue); Positive MIBG; Negative HVA/VMA

11p15 Epigenotype (BWSp) Profiles

Sample	SNP Testing	IC2 Methylation	IC1 Methylation	Summary
**Blood**	Normal chromosome 6	Normal	Normal	No 11p15 (OSH testing)
**Skin** (abdomen)	Normal Male	49.58% (N)	49.82% (N)	Normal 11p15 Mosaic Burden: <3%
**Pancreas** (normal)	~15% LOH 11p15.5p13	43.06% (LOM)	60.80% (GOM)	+11p15 (pUPD) Mosaic Burden: 17.74%
**Pancreas** (affected)	~45% LOH 11p15.5p13	25.30% (LOM)	72.17% (GOM)	+11p15 (pUPD) Mosaic Burden: 46.87%
**Thoracic Mass** (NBL)	Near triploid range	37.43% (LOM)	62.77% (GOM)	+11p15 abnormality Mosaic Burden: 25.34%

**Patient #03 (Female)**	**Characteristics and Phenotype Profiles**
**Prenatal Profile:**	MZ-triplets (natural); TRAP sequence with acardiac fetus (proband=donor)<br>Proband with severe BWSp (+IC2 LOM); Polyhydramnios; Twin – No Issues
**Birth Outcome:**	Born at GA=27 3/7 due to PPROM; Two live born female twins with discordant BWSp phenotypes (concordant blood); Placental Mesenchymal Dysplasia (PMD) diagnosed through pathology
**BWSp Phenotype and Craniofacial Profile**	Giant Omphalocele (entire liver extra-abdominal) Nephromegaly<br>Macroglossia (+TR); Cleft palate; Prominent occiput; Dysmorphic appearance<br>**BWSp Clinical Score = 8 points;** Features Not Present: Hypoglycemia, LO
**Other Notable Features:**	Cardiac anomalies (Large VSD detected prenatally); CDH (congenital diaphragmatic hernia); Tracheostomy required
**NBL Tumor Presentation:**	Incidental Detection (CT scan for other medical issue); Positive MIBG; Elevated HVA/VMA

11p15 Epigenotype (BWSp) Profiles

Samples	SNP Testing	IC2 Methylation	IC1 Methylation	Summary
Amniocytes	Normal Female	18.94% (LOM)	53.80% (N)	+11p15 (IC2 LOM) Mosaic Burden: 62.12%
Blood	Normal Female	23.75% (LOM)	49.42% (N)	+11p15 (IC2 LOM) Mosaic Burden: 52.50%
Tongue	NP	17.39% (LOM)	50.07% (N)	+11p15 (IC2 LOM) Mosaic Burden: 65.22%

BWSp Phenotype and Epigenotype Profile Established in Twin of Patient #03

**#03 Twin BWSp Phenotype:**	Transient hypoglycemia, Minor Abdominal Wall Defect – Umbilical Hernia

**BWSp Clinical Score = 4 points** *(shared PMD-placental mesenchymal dysplasia)*				
**Samples**	**SNP Testing**	**IC2 Methylation**	**IC1 Methylation**	**Summary**
Amniocytes	Normal Female	NP	NP	Not suspected for BWSp prenatally
Blood	Normal Female	23.81% (LOM)	50.50% (N)	+11p15 (IC2 LOM)<br>Mosaic Burden: 52.38%

Abbreviations:

BWSp=Beckwith-Wiedemann Spectrum; CT=Computed tomography; FNS=facial nevus simplex; GA=Gestational age (weeks); GOM=Gain of methylation (hypermethylation); HI=Hyperinsulinism; HVA/VMA=homovanillic acid/vanillylmandelic acid; IC1=Imprinting Control region 1 (*H19*/*IGF2*:IG-DMR); IC2=Imprinting Control region 2 (*KCNQ1OT1*:TSS-DMR); ILO=Isolated Lateralized Overgrowth; LGA=Large for gestational age (>2 standard deviations above mean); LO=Lateralized Overgrowth; LOM=Loss of Methylation (hypomethylation); MIBG=meta-iodobenzylguanidine; MZ=Monozygotic; N=normal; NP=not performed; OSH=outside hospital/laboratory; SNP=single nucleotide polymorphism; TR=tongue reduction; TRAP=Twin Reversed Arterial Perfusion Sequence; VSD=ventricular septal defect.
